# Automated evaluation of basketball skills using multimodal data fusion for improving shooting training efficiency

**DOI:** 10.1016/j.isci.2026.117915

**Published:** 2026-10-15

**Authors:** Puzi Sun

**Affiliations:** 1 Department of Physical Education, Anhui Traffic Vocational and Technical College, Hefei, Anhui 230051, China

**Keywords:** multimodal fusion, basketball shooting, skill assessment, training feedback, motor learning, sports analytics

## Abstract

Reliable basketball shooting assessment requires integration of complementary movement signals and actionable feedback. This study developed the multimodal shooting evaluation and efficiency model (MSEEM) and evaluated it in 156 players assigned to MSEEM feedback or standard coaching. RGB video, pose, inertial measurement unit signals, ball trajectories, and shot outcomes were fused to estimate rubric-based total and component scores. MSEEM achieved lower mean absolute error (4.82 ± 0.31) and stronger rank agreement (*ρ* = 0.824) than SlowFast (6.24 ± 0.41; *ρ* = 0.702). MSEEM feedback also produced steeper training improvement and higher 14-day retention (*d* = 0.68), supporting standardized component-targeted feedback.

## Highlights

• MSEEM fuses RGB, pose, IMU, trajectory, and outcome signals for shooting assessment
• MSEEM reduced rubric-score prediction error versus video-only SlowFast
• Component-targeted feedback accelerated improvement across six training sessions
• MSEEM feedback improved 14-day retention under standardized practice conditions

## Graphical abstract

**Figure:**
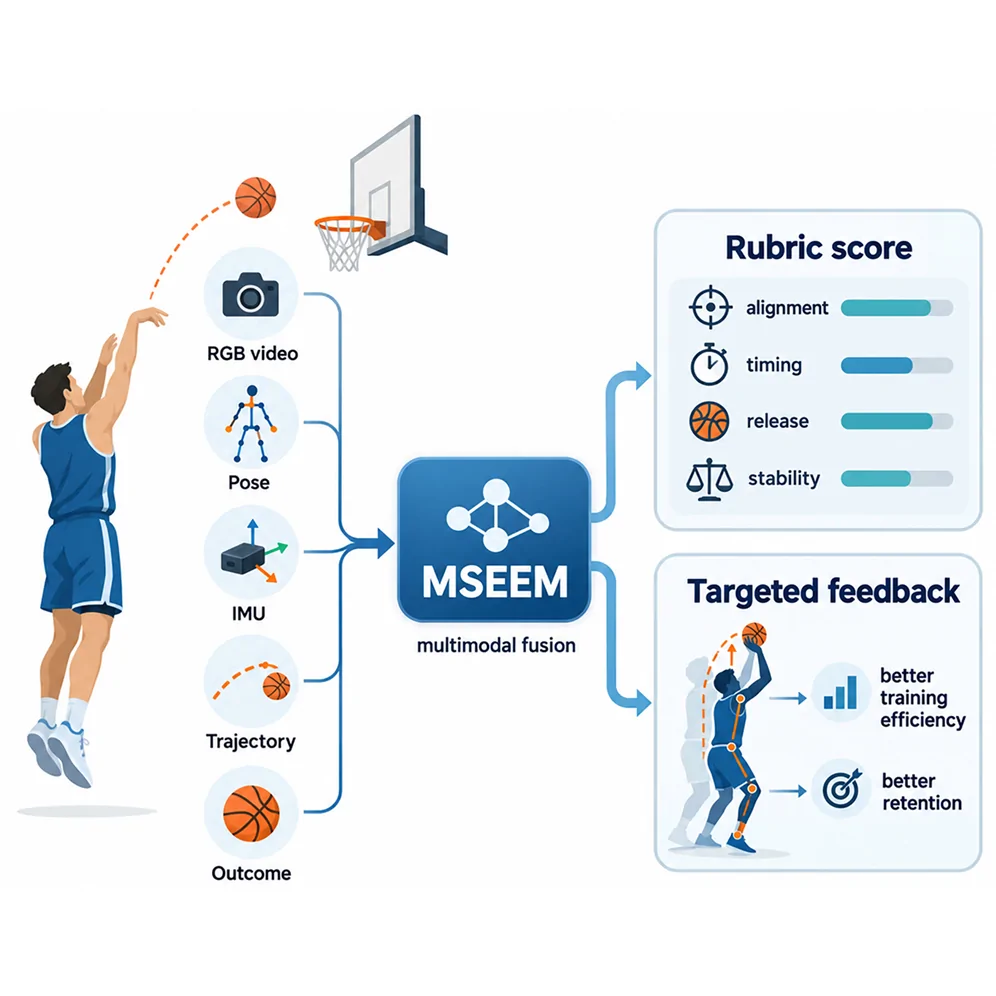

## Teaser

Procedure; Health promotion

## Introduction

Evaluation of basketball shooting technique requires structured assessment because scoring can be affected by occlusion, changes in viewing perspective, and defender proximity.^1^ Shooting technique also varies across attempts and conditions; consequently, technical quality should not be inferred from a single make-or-miss outcome.^2^ Rubric-based assessment provides a 0–100 shooting-skill score while retaining component-level information that can identify correctable technical errors and track improvement across sessions.^3^ Multimodal assessment integrates synchronized red-green-blue (RGB) video for contextual information, markerless pose keypoints for joint geometry, inertial measurement unit (IMU) signals for wrist-forearm dynamics, ball-trajectory estimates for release and flight characteristics, and shot outcomes for task success.^4^ Multimodal fusion combines these complementary spatiotemporal streams to maintain informative technique scoring when an individual modality is unavailable, unreliable, or degraded by occlusion, noise, or incomplete observations.^5^

Existing automated approaches increasingly quantify basketball shooting mechanics, but their value for training depends on whether computational outputs can be translated into interpretable measures of technique. Sensor- and vision-based systems commonly emphasize recognition or prediction performance without consistently linking model outputs to specific technical components or controlled feedback outcomes.^6^ Coaching-oriented assessment instead requires interpretable measures of elbow positioning, release mechanics, jump-to-release timing, and follow-through stability that can be mapped to component and total rubric scores.^7^ Assessment across free throws, catch-and-shoot attempts, off-dribble shots, and lightly contested shots also provides broader evidence of shooting performance than stationary free throws alone by incorporating variation in movement preparation and task demands.^8^ Such evaluation therefore requires both technically meaningful scoring and evidence that the resulting measures remain informative across relevant shooting conditions.^9^

Clear separation of predictive and training-related outcomes is equally important when automated assessment is evaluated longitudinally. In this study, training efficiency denotes the rate of improvement in blinded coach-derived rubric score per session-minute across the six training sessions, whereas retention denotes rubric-score performance at the delayed re-test following training. Generalization refers specifically to predictive performance across held-out players, sessions, or environments and is distinct from retention, which concerns persistence of acquired performance over time. Measurement quality must also be considered because markerless kinematic estimates can vary with camera geometry, movement characteristics, and recording conditions.^10^ Evidence from markerless motion-capture validation further indicates that accuracy, validity, and reliability should be treated as distinct measurement properties and should not be inferred solely from predictive agreement.^11^ Independent coach-derived rubric labels, controlled feedback delivery, and clearly differentiated measures of generalization, training efficiency, and retention therefore provide the basis for evaluating whether automated shooting assessment can support reproducible and training-relevant performance evaluation.

### Multimodal shooting evaluation and training efficiency

Biomechanical evidence indicates that basketball shooting quality depends on coordinated whole-body posture and release control across interacting joint actions. Verhoeven and Newell^12^ compared skilled and less-skilled free-throw shooters using laboratory motion capture and associated higher proficiency with more stable postural organization and coordinated ball-release control. Nakano et al.^13^ similarly demonstrated hierarchical redundancy in free-throw kinematics, whereby compensatory coordination among the shoulder, elbow, and wrist helped preserve stable release conditions. Using markerless motion capture, Cabarkapa et al.^14^ associated shooting performance with knee-extension velocity, trunk stability, center-of-mass control, and release height, supporting the relevance of multiple coordinated biomechanical features. However, these studies did not jointly establish a rubric-grounded 0–100 scoring framework, component-level calibration across athletes and recording conditions, and experimentally controlled endpoints for training efficiency and delayed retention. This distinction is important because biomechanical description alone does not establish whether derived measurements can produce reproducible technique scores or feedback that supports longitudinal skill acquisition.

Automated movement-analysis systems have improved recognition and classification of sports actions, but their translation into coaching-relevant assessment remains limited. Khobdeh et al.^15^ demonstrated the value of computational approaches for sports-movement analysis, although the emphasis remained on recognition performance, with limited component-level technique evaluation. Liu et al.^16^ similarly advanced automated performance classification, but did not establish whether predictive gains translated into faster skill acquisition or delayed retention. Huang et al.^17^ proposed DESNet for real-time pose estimation using Internet of Things sensing and computer-vision data; however, the evaluated tasks were not specific to basketball shooting and longitudinal training outcomes were not examined. Chen et al.^18^ applied machine-learning models to athlete performance data for skill-level classification, supporting standardized assessment while providing limited information on which technical deficits should be corrected during training. In basketball-specific research, Hsu et al.^19^ developed PoseShot for fine-grained free-throw action recognition and reported stronger recognition performance than comparator architectures, but recognition accuracy does not establish agreement with an independent coach-derived technique rubric or demonstrate improvement in repeated training. Seino et al.^20^ further combined video and spatiotemporal movement features to generate expert-oriented feedback, although longitudinal transfer and delayed retention were not established. Existing evidence therefore supports automated recognition, pose estimation, and feedback generation, but remains fragmented across predictive accuracy, interpretable component scoring, cross-player or cross-environment generalization, and experimentally measured training efficiency and retention.

Multimodal fusion can reduce failure modes associated with individual sensors, although much of the existing evidence concerns activity recognition or pose recovery, with limited focus on rubric-based basketball shooting assessment.^21^^,^^22^ Dong et al.^23^ evaluated feature-level and decision-level fusion of RGB video and inertial streams and reported greater robustness to occlusion and visual noise than video-only analysis, but the outputs were not mapped to calibrated component-level technique scores. Xefteris et al.^24^ combined camera and IMU signals within a hybrid temporal modeling framework for three-dimensional pose estimation and demonstrated improved robustness under restricted viewpoints, although basketball shooting under defender interference was not evaluated. Khan et al.^25^ applied temporal transformer fusion to visual and skeletal streams to model long-range dependencies, but the validation setting did not capture the full variation associated with dynamic shooting conditions, court lighting, or camera geometry. These findings support multimodal representation as a means of improving robustness, while leaving unresolved whether synchronized modalities can improve agreement with an independent shooting rubric across held-out players, sessions, and environments. Within the present framework, that unresolved problem concerns generalization of scoring performance, which remains analytically distinct from training efficiency during the intervention and retention at delayed re-testing.

Augmented feedback can support motor learning when performance errors are communicated with controlled timing, dosage, and task relevance, although the persistence of these effects varies across populations and intervention designs.^26^^,^^27^ Ueyama and Harada^28^ used augmented-reality visualization of an optimal ball trajectory during novice free-throw practice and reported improved shooting accuracy; however, the intervention was short and delayed retention was not evaluated comprehensively. He and Wei^29^ found that real-time augmented feedback improved skill acquisition, motivation, and retention in youth team-sport training, but the activities were not specific to basketball shooting and the contribution of individual feedback components was not isolated. Ma et al.^30^ reported improved movement quality and engagement with pose-recognition feedback in physical education, although competitive transfer and blinded outcome assessment were not clearly established. These studies indicate that rapid, technique-relevant feedback can enhance motor learning, but evidence remains limited regarding whether such feedback can be grounded in independently rated shooting components, maintain scoring performance across players and recording conditions, and produce sustained improvements in training efficiency and delayed retention.

### Theoretical and analytical framework

Rubric-grounded component scoring, cross-player and cross-environment reliability, and controlled evidence linking automated feedback to training efficiency and delayed retention remain insufficiently integrated in existing basketball shooting assessment. The Multimodal Shooting Evaluation and Efficiency Model (MSEEM) was developed to address this limitation through a unified scoring-and-feedback framework that combines synchronized red-green-blue (RGB) video for scene context and defender proximity, markerless pose keypoints for joint geometry, inertial measurement unit (IMU) signals for wrist-forearm dynamics, ball-trajectory features for release mechanics, and shot outcome as a separate indicator of task success. These streams are fused to estimate a 0–100 shooting-skill score together with four component subscores representing alignment, timing, release mechanics, and stability. Independent coach ratings on a structured rubric provide the reference standard, while model performance is evaluated across cross-player, cross-session, and cross-environment conditions. The resulting scores are also linked to a fixed feedback protocol that delivers the overall score and the two highest-priority correctable components after each 10-shot block.

MSEEM connects multimodal measurement, interpretable component scoring, generalization testing, and experimentally controlled feedback within a single analytical framework. Previous studies have largely focused on action recognition, pose estimation, isolated biomechanical variables, or short-term feedback effects, providing limited evidence that computational performance translates into reproducible technique scoring and sustained learning outcomes. MSEEM advances this evidence base by grounding predictions in independently rated rubric scores, preserving component-level interpretability, evaluating robustness across players, sessions, and environments, and separating predictive generalization from intervention outcomes. The framework further links scoring fidelity to a randomized feedback intervention in which training efficiency is quantified as rubric-score improvement per session-minute and retention is assessed at delayed re-testing. This integration enables evaluation of whether gains in multimodal technique representation translate into measurable improvements in learning rate and persistence of performance.

### Objectives

The objectives of the study are (1) to develop a multimodal framework for automated basketball shooting evaluation that integrates visual, kinematic, inertial, trajectory, and shot-outcome information into rubric-based total and component-level assessments; (2) to determine whether multimodal integration provides more robust and consistent shooting-skill evaluation than single-modality and reduced-modality approaches across players, sessions, and recording environments; and (3) to determine whether standardized component-targeted feedback generated from the shooting assessment improves training efficiency and delayed retention compared with standard coaching feedback. The following research questions guide the study. RQ1: Does multimodal integration provide more robust and consistent assessment of basketball shooting technique than single-modality and reduced-modality approaches across players, sessions, and recording environments? RQ2: Does component-targeted MSEEM feedback improve training efficiency and delayed retention compared with standard coaching feedback? The corresponding hypotheses are: H1: Multimodal integration will provide more robust and consistent basketball shooting assessment than single-modality and reduced-modality approaches across players, sessions, and recording environments. H2: Participants receiving component-targeted MSEEM feedback will demonstrate greater training efficiency and stronger delayed retention than participants receiving standard coaching feedback.

## Results

### Baseline features

Baseline characteristics were comparable between conditions (Figure S3 and Tables 1, 2, and 3), including age, sex, playing level, rubric scores, and shot-type make rates. Training adherence was complete in both conditions: all participants completed six scheduled sessions and 72 training blocks, and all scheduled block-level feedback episodes were delivered. All 156 participants completed the immediate post-test and 14-day retention assessment, with no post-assignment exclusions, missing primary outcomes, or training-related adverse events.

**Table 1:** Feature set and computation parameters used for MSEEM inputs

Modality stream	Feature family	Specific feature (unit)	Computation window/parameters
Red-green-blue video	context and visibility	keypoint visibility ratio (%)	% frames with mean pose confidence ≥0.50 in −600 to +800 ms window
	context and viewpoint	occlusion proxy (unitless)	1 − keypoint visibility ratio; computed per shot and averaged per block
Markerless pose	alignment	elbow flexion at release (°)	angle(shoulder-elbow-wrist) at *t* = 0 ms
	alignment	shoulder flexion at release (°)	3D sagittal-plane angle between the trunk-shoulder and shoulder-elbow vectors at *t* = 0 ms
	alignment	trunk forward lean at release (°)	3D trunk-axis angle relative to the global vertical axis at *t* = 0 ms
	timing	jump-to-release interval (ms)	time between peak hip vertical velocity and *t* = 0 ms
	stability	follow-through variance (°)	variance of wrist and elbow angles in +100 to +600 ms window
	smoothness	jerk index (m/s^3^)	root-mean-square jerk of wrist keypoint in −300 to +300 ms window
Inertial measurement unit	timing	peak wrist angular velocity (°/s)	peak gyroscope magnitude in −200 to +200 ms window
	timing	time-to-peak angular velocity (ms)	time from −200 ms to peak within −200 to +200 ms
	release dynamics	angular velocity impulse (°)	∫
	smoothness	acceleration variability (m/s^2^)	standard deviation of acceleration magnitude in −300 to +300 ms
Ball trajectory	release mechanics	release angle (°)	initial 3D ball-velocity angle over the first 120 ms after release.
	release mechanics	apex height (m)	maximum reconstructed ball z-coordinate between release and rim-plane crossing.
	release mechanics	entry angle (°)	descending 3D ball-velocity angle at intersection with the 3.05-m rim plane
Shot outcome	task success	make indicator (0/1)	logged per shot; aggregated as make rate per block (%)
Block aggregation	consistency	component variability (unit-matched)	within-block standard deviation for each shot-level feature
	efficiency support	block time (s)	time from first to last shot in the block, excluding rests

**Table 2:** Training configuration and hyperparameters applied uniformly

Category	Parameter	Value/setting
Data windowing	shot window	−600 ms to +800 ms relative to release
Sampling	unified timebase	200 Hz (interpolated where needed)
Filtering	pose smoothing	4th-order zero-lag Butterworth, 10 Hz cutoff
Filtering	inertial smoothing	4th-order zero-lag Butterworth, 20 Hz cutoff
Normalization	feature scaling	*Z* score using training-set statistics only
Train/validation/test	split scheme	five-fold participant-grouped cross-player evaluation; three leave-one-environment-out evaluations; frozen-model evaluation across sessions 1–6. Preprocessing and tuning parameters estimated from training data only
Batch size	mini-batch	64 shot windows
Epochs	maximum epochs	80
Early stopping	patience	10 epochs on validation mean absolute error
Optimizer	optimizer	AdamW, weight decay 1 × 10^−4^
Learning rate	initial learning rate	3 × 10^−4^ with 5-epoch warmup; cosine decay thereafter
Regularization	dropout	0.20 in encoders and fusion layer
Loss	total score loss	Huber loss, *δ* = 1.0
Loss	component losses	mean squared error per component head
Loss weighting	total:components	1.0 : 0.25 each (alignment, timing, release, stability)

**Table 3:** Baseline participant characteristics and intervention adherence by study condition

Variable	MSEEM-feedback	Control coaching	*p* value
Age (years)	21.4 ± 2.3	21.6 ± 2.4	0.598
Sex, male n (%)	52 (66.7%)	50 (64.1%)	0.728
Playing level, competitive n (%)	33 (42.3%)	35 (44.9%)	0.742
Years of playing experience (years)	5.2 ± 2.1	5.0 ± 2.2	0.564
Dominant shooting hand, right n (%)	69 (88.5%)	68 (87.2%)	0.808
Baseline rubric total score (0–100)	62.8 ± 8.9	62.1 ± 9.1	0.633
Alignment subscore (0–25)	15.7 ± 2.6	15.5 ± 2.7	0.642
Timing subscore (0–25)	15.3 ± 2.5	15.4 ± 2.6	0.811
Release mechanics subscore (0–25)	15.9 ± 2.7	15.6 ± 2.8	0.505
Stability subscore (0–25)	15.8 ± 2.4	15.6 ± 2.5	0.626
Free-throw make rate (%)	63.5 ± 10.2	62.8 ± 10.5	0.676
Catch-and-shoot make rate (%)	51.2 ± 11.8	50.1 ± 12.1	0.561
Off-dribble make rate (%)	44.7 ± 10.6	45.3 ± 10.4	0.718
Lightly contested make rate (%)	38.4 ± 9.9	37.6 ± 10.1	0.620
Baseline familiarization exposure (min)	18.9 ± 3.8	19.1 ± 3.6	0.734
Training sessions completed per participant, median (IQR)	6 (6–6)	6 (6–6)	1.000
Scheduled participant-sessions completed, n/N (%)	468/468 (100%)	468/468 (100%)	1.000
Training blocks completed per participant, median (IQR)	72 (72–72)	72 (72–72)	1.000
Scheduled training blocks completed, n/N (%)	5,616/5,616 (100%)	5,616/5,616 (100%)	1.000
Scheduled block-level feedback episodes delivered, n/N (%)	5,616/5,616 (100%)	5,616/5,616 (100%)	1.000
Immediate post-test completed, n/N (%)	78/78 (100%)	78/78 (100%)	1.000
14-day retention completed, n/N (%)	78/78 (100%)	78/78 (100%)	1.000
Training-related adverse events, n (%)	0 (0%)	0 (0%)	1.000

### Primary outcomes

Primary model comparisons used corrected paired resampling with Holm-adjusted *p* values (Table 4). MSEEM achieved an MAE of 4.82 ± 0.31 compared with 6.24 ± 0.41 for SlowFast, corresponding to an adjusted paired difference of −1.42 points, 95% CI [−2.05, −0.79], t(4) = 6.22, *p* = 0.008. Spearman ρ was 0.824 ± 0.019 versus 0.702 ± 0.031, with a paired difference of 0.122, 95% CI [0.074, 0.170], *p* = 0.009. Removing pose increased MAE to 5.79 ± 0.39, with a paired difference of −0.97 points, 95% CI [−1.50, −0.44], t(4) = 5.08, *p* = 0.012.

**Table 4:** Total-score prediction performance

Model	MAE	Spearman rho	Calibration intercept	Calibration slope
MSEEM	4.82 ± 0.31	0.824 ± 0.019	0.41 ± 0.33	0.98 ± 0.04
Ablation without RGB video	5.36 ± 0.34	0.781 ± 0.023	0.62 ± 0.39	0.93 ± 0.05
Ablation without pose	5.79 ± 0.39	0.752 ± 0.025	0.78 ± 0.46	0.90 ± 0.06
Ablation without IMU	5.55 ± 0.33	0.763 ± 0.022	0.71 ± 0.42	0.91 ± 0.05
Ablation without ball trajectory	5.44 ± 0.32	0.771 ± 0.020	0.67 ± 0.41	0.92 ± 0.05
Ablation without outcome input	4.95 ± 0.29	0.816 ± 0.018	0.39 ± 0.34	0.99 ± 0.04
Ablation concatenation fusion	5.12 ± 0.30	0.803 ± 0.021	0.55 ± 0.36	0.95 ± 0.05
Comparator SlowFast video-only	6.24 ± 0.41	0.702 ± 0.031	1.06 ± 0.52	0.86 ± 0.07
Comparator TimeSformer video-only	6.03 ± 0.38	0.721 ± 0.028	0.98 ± 0.49	0.88 ± 0.07
Comparator Video Swin Transformer video	5.82 ± 0.36	0.736 ± 0.026	0.91 ± 0.47	0.89 ± 0.06
Comparator ST-GCN pose-only	6.48 ± 0.45	0.681 ± 0.033	1.18 ± 0.55	0.84 ± 0.08
Comparator DeepConvLSTM IMU-only	6.72 ± 0.48	0.664 ± 0.034	1.25 ± 0.57	0.82 ± 0.09
Comparator IMU Transformer IMU-only	6.55 ± 0.46	0.676 ± 0.032	1.20 ± 0.56	0.83 ± 0.08
Comparator outcome-only	7.10 ± 0.52	0.612 ± 0.041	1.42 ± 0.62	0.78 ± 0.10

The prespecified mixed-effects analysis identified a positive MSEEM-feedback × time coefficient (Figure 1), with *B* = 0.037, SE = 0.012, 95% CI [0.013, 0.061], *t* = 3.080, *p* = 0.003. The adjusted time effect was also positive, with *B* = 0.102, SE = 0.031, 95% CI [0.041, 0.163], *t* = 3.290, *p* = 0.001.

**Figure 1:**
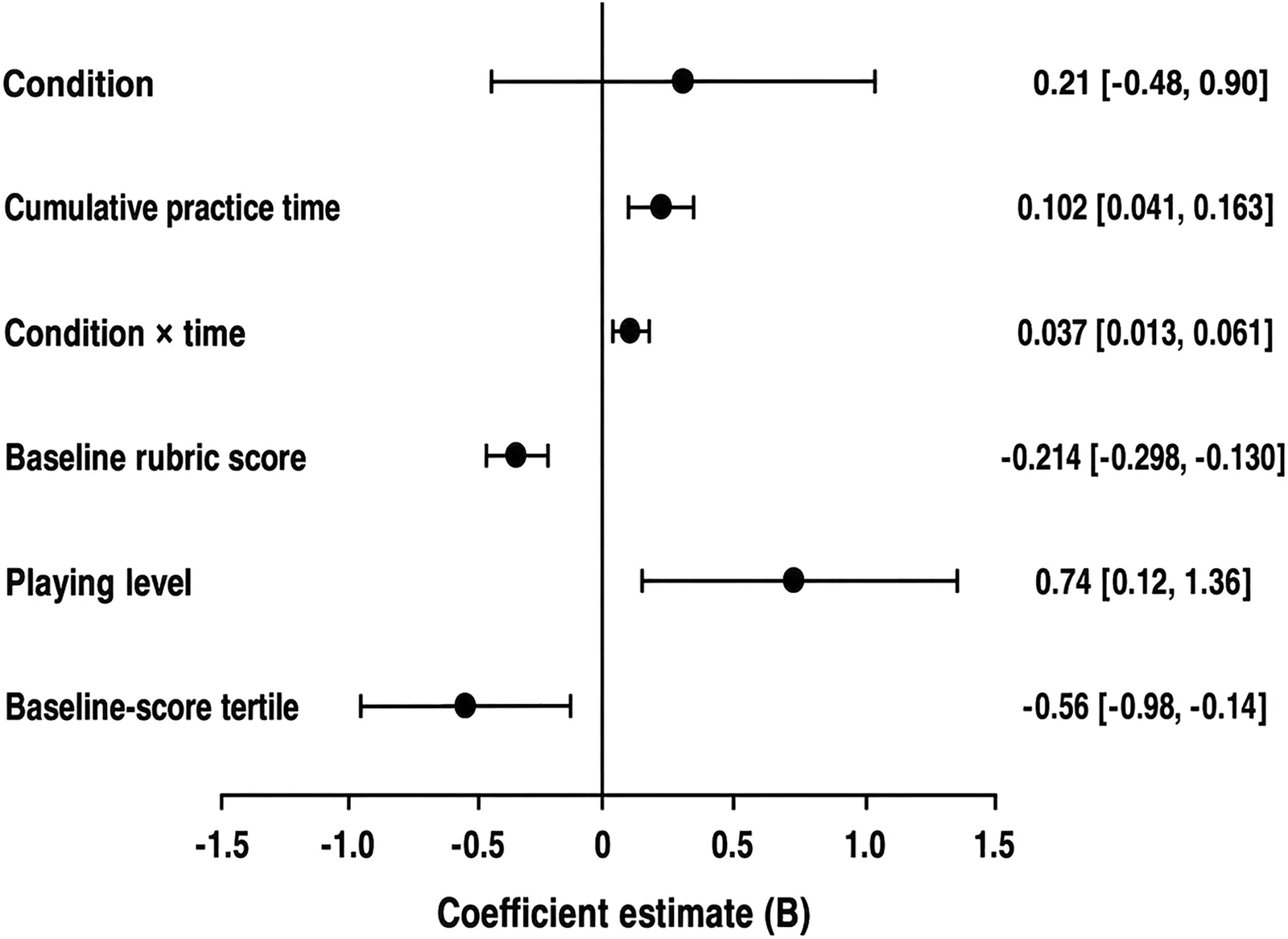
Adjusted fixed-effect estimates from the training-efficiency mixed-effects model Points represent regression coefficients and horizontal bars represent 95% confidence intervals for condition, cumulative practice time, condition × time, baseline rubric score, playing level, and baseline-score tertile. The model included 156 participants and was estimated using restricted maximum likelihood with Satterthwaite approximation.

Outcome-stream sensitivity was limited despite the predictive association between shot success and rubric score. Flipping the binary outcome while holding all other inputs fixed changed the predicted total score by 0.63 ± 0.18 points on average, with a 95th percentile of 1.31 points and a maximum change of 1.88 points. Mean gated-attention weights were 0.226 for RGB, 0.267 for pose, 0.231 for IMU, 0.205 for ball trajectory, and 0.071 for outcome. The small outcome weight and limited counterfactual score displacement were consistent with the modest MAE increase from 4.82 to 4.95 when outcome input was removed (Table 4).

The adjusted retention contrast favored MSEEM feedback (Figure 2), with *B* = 5.90, SE = 1.89, 95% CI [2.17, 9.63], t(148) = 3.120, *p* = 0.002, and *d* = 0.68. Descriptive retention scores were 70.4 ± 8.5 for MSEEM feedback and 64.1 ± 8.9 for control coaching. The pooled retention-score standard deviation used for standardization was 8.70 points.

**Figure 2:**
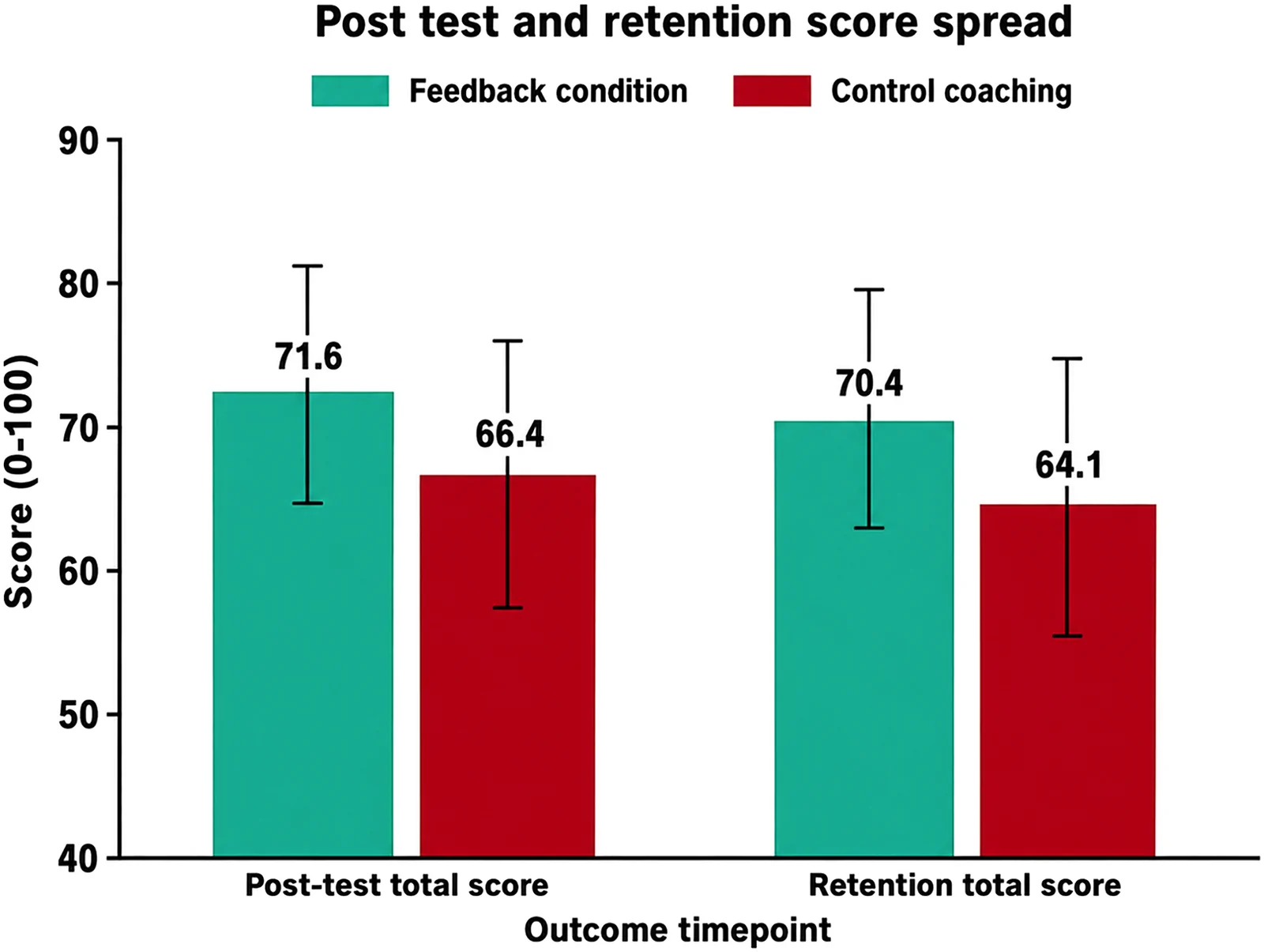
Post-test and retention total rubric scores for MSEEM-feedback and control coaching conditions Bars represent group means and error bars represent standard deviations; *n* = 78 participants per condition. The adjusted retention contrast was estimated using the prespecified covariate-adjusted mixed-effects model.

### Secondary outcomes

Component-score prediction performance (Table S5) for MSEEM showed alignment MAE of 1.12 ± 0.07, Spearman ρ of 0.812 ± 0.022, and calibration slope of 0.99 ± 0.05, with stability MAE of 1.10 ± 0.07, ρ of 0.820 ± 0.021, and slope of 0.98 ± 0.05. The outcome-only comparator showed an alignment MAE of 1.88 ± 0.15 with a slope of 0.78 ± 0.10 and a stability MAE of 1.90 ± 0.15 with a slope of 0.77 ± 0.10.

Component-level MAE was lowest for MSEEM across alignment, timing, release mechanics, and stability (Figure 3). Removal of RGB video or pose increased error across the component scores, while the selected external comparators showed higher MAE across all four components. A covariate-adjusted mixed-effects model estimated shot-type-specific training effects from condition, time, condition × time, baseline score, baseline tertile, and playing level (Table S6). The lightly contested condition showed a positive MSEEM-feedback × time effect, *B* = 0.048 ± 0.015, 95% CI [0.019, 0.077], *t* = 3.260, Holm-adjusted *p* = 0.004, and a positive retention contrast, *B* = 7.30 ± 2.18, 95% CI [3.00, 11.60], *t* = 3.350, Holm-adjusted *p* = 0.004.

**Figure 3:**
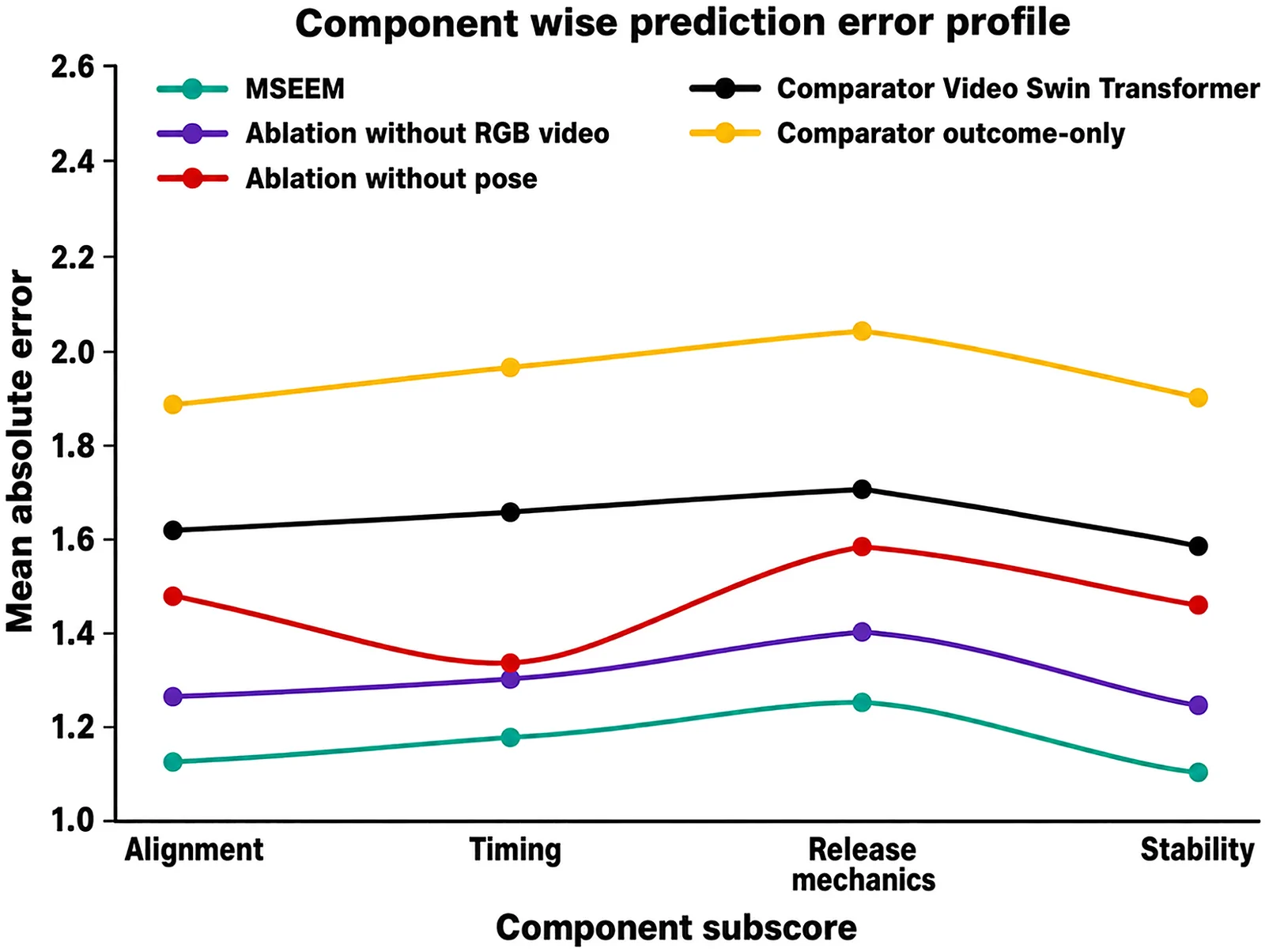
Component-wise mean absolute error profile across alignment, timing, release mechanics, and stability for MSEEM, selected ablations, and external comparators Values summarize component-level prediction error across the five participant-grouped outer folds.

Positive MSEEM-feedback × time estimates were observed across all four shot types, with the largest estimate and retention contrast for lightly contested shots (Figure 4A). Alignment and stability showed the largest component-level time and follow-up estimates, while release mechanics showed smaller coefficients (Figure 4B). A covariate-adjusted mixed-effects model estimated component-level change from condition, time, condition × time, baseline score, baseline tertile, and playing level (Table S7). Stability showed a positive MSEEM-feedback × time effect, *B* = 0.013 ± 0.004, 95% CI [0.005, 0.021], *t* = 3.250, Holm-adjusted *p* = 0.004.

**Figure 4:**
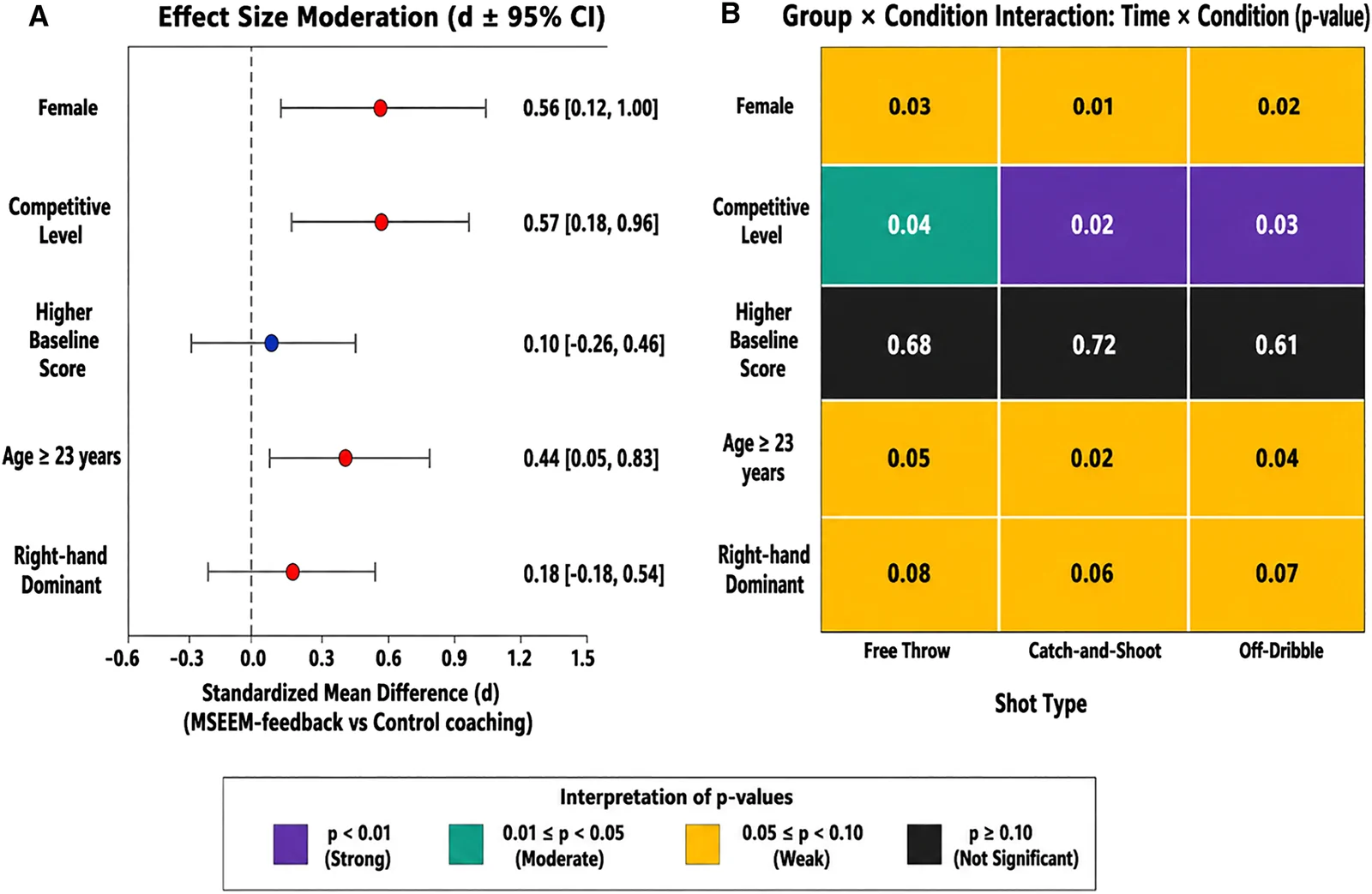
Shot-type and component-specific training effects across intervention and retention assessments (A) Shot-type training-effect estimates for the MSEEM-feedback × time term with corresponding retention contrasts across free throw, catch-and-shoot, off-dribble, and lightly contested shots. Points and bars represent adjusted coefficients and 95% confidence intervals. (B) Component training-effect coefficient map summarizing time-term, post-test contrast, and retention-contrast estimates.

### Robustness across baseline tertiles and playing level

A mixed-effects moderation analysis was conducted to test whether baseline tertile and playing level modified the MSEEM-feedback time effect. No statistically significant baseline tertile upper × MSEEM-feedback × time interaction was observed (*B* = −0.004, SE = 0.011, 95% CI [−0.026, 0.018], *t* = −0.360, *p* = 0.719). Moderation intervals cluster near zero with confidence ranges spanning both directions across baseline tertile and playing-level terms, indicating limited evidence of differential time-slope effects by these moderators within the fitted model (Figure S4A). Retention-contrast moderator intervals are similarly centered near zero with wide uncertainty ranges, indicating that subgroup adjustment terms contribute small additional effects relative to the overall retention difference (Figure S4B). Normalized moderator directions were positive for the middle baseline tertile and competitive playing level and negative for the upper baseline tertile across the time-interaction and retention contrasts (Figure S5A). The corresponding pathway map summarizes the direction and relative magnitude of these exploratory moderator estimates (Figure S5B).

## Discussion

The present study showed that MSEEM combined multimodal shooting information into a rubric-grounded scoring framework that achieved stronger agreement with coach-derived scores and supported more effective training feedback under standardized practice conditions. The model maintained lower prediction error, stronger rank agreement, and near-unit calibration across the prespecified generalization analyses, while the feedback condition produced a steeper training improvement trajectory and higher delayed retention than standard coaching. These findings directly addressed RQ1 by demonstrating that multimodal fusion improved rubric-score prediction across cross-player, cross-session, and cross-environment evaluations, thereby supporting H1. They also addressed RQ2 by showing that standardized component-targeted feedback delivered after each 10-shot block improved training efficiency and 14-day retention, supporting H2. The effect magnitudes were substantial, including *d* = 2.78 for the principal between-model comparison and *d* = 0.68 for the adjusted retention contrast, while fold-specific calibration slopes remained within 0.02 of unity. These magnitudes are comparatively large in relation to prior model-comparison and feedback-training literature and should therefore be interpreted within the controlled acquisition, practice, and feedback conditions used in the study.

The current study shows that multimodal fusion produced more stable rubric-score prediction across players, sessions, and recording environments, while the outcome stream contributed only a limited share of the final estimate. Xefteris et al.^24^ reported that combining visual and inertial information improved three-dimensional pose representation under challenging viewpoints, which supports the present advantage of retaining complementary motion streams. A comparable pattern was reported by Dong et al.,^23^ where inertial and video fusion reduced sensitivity to occlusion and visual noise, consistent with the degradation observed when core MSEEM modalities were removed. These comparisons suggest that robust shooting evaluation depends on distributing relevant information across contextual, geometric, temporal, and trajectory representations. RGB and pose features characterize spatial organization, IMU signals preserve release-phase dynamics, and ball-trajectory features provide complementary information about shooting execution. The small contribution of the outcome stream further indicates that technique-related information carried most of the predictive signal, with make-or-miss status contributing comparatively little. This multimodal structure is consistent with stronger generalization because informative streams can compensate for localized degradation in individual inputs, although agreement remains specific to the coach-derived reference standard used in the study.

The analysis suggests that component-targeted feedback supported more efficient improvement by converting rubric-based estimates into stable corrective priorities during repeated shooting practice. Seino et al.20 reported that movement features aligned with expert assessment can support technically specific feedback, which is consistent with the present use of component deficits to guide correction. Deng et al.^8^ showed that vision and pose information can recover interpretable characteristics of basketball shooting, supporting the use of structured movement representations as a basis for feedback selection. Within MSEEM, separate estimates of alignment, timing, release mechanics, and stability allowed feedback to focus consistently on the most pronounced technical deficits after each block. This organization provides a plausible instructional advantage because it limits competing cues and preserves continuity in the correction target across practice repetitions. Repeated prioritization of the largest identified component deficits may facilitate more focused adjustment and reduce variability in the corrective information presented to the learner. Attention allocation and cognitive processing were not measured directly, so this explanation should be treated as an interpretation of the observed training pattern because the proposed psychological mechanism was not directly tested.

The observed pattern reveals that the training advantage persisted at delayed assessment, indicating that the standardized feedback protocol supported performance beyond the immediate practice period. Hsu et al.^19^ found that biomechanically informed pose architectures can improve the consistency of basketball shooting assessment, which supports the value of stable technique representation across repeated evaluations. Rapid visual feedback was also associated with improved shooting practice in the study by Ueyama and Harada,^28^ aligning with the present use of short-latency feedback after predefined shot blocks. The present contribution extends this evidence by linking multimodal rubric estimation, fixed component prioritization, and delayed retention within the same controlled training framework. A plausible explanation is that consistent component estimates generated repeatable correction targets across sessions, allowing technical adjustments to be reinforced under a stable feedback rule. The retention effect therefore supports continuity between assessment and correction, where stable scoring provides consistent technical priorities that can be repeatedly practiced and consolidated across sessions. Within MSEEM, this linkage strengthens the functional connection between multimodal evaluation and retained shooting performance by maintaining alignment between identified deficits, corrective cues, and subsequent shooting practice.

### Theoretical and practical implications

The findings extend multimodal skill-evaluation theory by showing that alignment, timing, release mechanics, and stability can be represented as coordinated dimensions of shooting quality within a rubric-grounded assessment framework. The contribution lies in linking complementary movement representations with a structured reference standard and then converting component estimates into predefined corrective cues. This supports a learning-oriented view of automated evaluation in which predictive agreement, component specificity, feedback timing, and repeated practice operate as connected elements of the assessment-feedback process. The practical value of MSEEM therefore concerns its capacity to provide standardized, coach-interpretable feedback during controlled shooting practice while preserving consistent scoring logic across sessions. Its use can support progress monitoring and identification of technical priorities within structured training environments. These implications concern agreement with the coach-derived rubric used in this study and should not be interpreted as evidence that the scores constitute universally objective or context-independent measures of shooting quality.

The study demonstrates that multimodal fusion can improve predictive agreement with coach-derived basketball shooting scores across the evaluated generalization conditions and that these component-level estimates can support effective feedback when delivered through a standardized training protocol. Integration of RGB, pose, IMU, and ball-trajectory information provided complementary representations of alignment, timing, release mechanics, and stability, while the limited contribution of the outcome stream indicates that scoring was driven primarily by technique-related information. The training experiment further showed that rapid component-targeted feedback was associated with greater training efficiency and stronger 14-day retention under controlled practice conditions. These findings establish MSEEM as a proof-of-concept assessment-feedback framework for standardized basketball shooting training. Broader application in academies, schools, independent facilities, and competitive environments requires external validation under variable camera geometries, active defensive pressure, occlusion, multi-player interaction, and independent coaching settings.

### Limitations of the study

Several limitations define the scope of these findings. First, testing and training were conducted under standardized indoor conditions with fixed camera positions, controlled shooting stations, standardized lighting, and predetermined feedback timing. The lightly contested condition used a passive defender positioned 0.75 m from the shooter, which provided controlled proximity interference but did not reproduce the spatial pressure, movement, decision demands, and occlusion encountered during active competition. Transfer to competitive game performance therefore remains unestablished. Second, the reference scores were derived from a predefined coach rubric. Blinded assessment and strong inter-rater agreement improved consistency, although the reference standard may still reflect rubric-specific coaching judgments and does not establish universal measurement validity. Third, the control group received standard coaching without a visually matched sham tablet condition. Consequently, the contribution of component-specific information cannot be fully separated from possible motivational, expectancy, or engagement effects associated with receiving structured digital feedback. Fourth, the moderation analyses were exploratory and were not powered specifically for subgroup interaction effects; the broad retention-contrast intervals and estimates near zero indicate considerable uncertainty regarding effect modification by baseline skill and playing level. Future research should evaluate MSEEM across independent sites, alternative camera geometries, variable lighting, active defenders, greater occlusion, multi-player sequences, and game-derived shooting episodes. Additional studies should also test visually matched control feedback, longer retention intervals, alternative feedback schedules, and uncertainty-aware scoring under degraded sensing conditions.

## Resource availability

### Lead contact

Further information and requests for resources should be directed to and will be fulfilled by the lead contact, Puzi Sun (summer3633@126.com).

### Materials availability

This study did not generate new unique reagents or biological materials.

### Data and code availability

• Data: The data supporting the findings of this study are publicly available in Zenodo at https://doi.org/10.5281/zenodo.22828547. Supporting tables and figures are provided in the supplemental information accompanying this article.
• Code: Reproducibility analysis code corresponding to the reported model performance, cross-validation, outcome-sensitivity, training-outcome, shot-type, component-level, and moderation analyses is publicly available in Zenodo at https://doi.org/10.5281/zenodo.22828547.
• Other: Any additional information required to reanalyze the data reported in this paper is available from the lead contact upon request.

## Acknowledgments

The author thanks the participating basketball players and coaches for their contribution to the study. This study received no external funding.

## Author contributions

P.S.: conceptualization, methodology, investigation, data curation, formal analysis, visualization, writing – original draft, and writing – review and editing.

## Declaration of interests

The authors declare no competing interests.

## STAR★Methods

### Key resources table

**Table:** 

REAGENT or RESOURCE	SOURCE	IDENTIFIER
**Deposited data**		

Raw multimodal basketball-shooting dataset supporting this study	This paper	Zenodo: https://doi.org/10.5281/zenodo.22828547

**Software and algorithms**		

Reproducibility analysis code for MSEEM model-performance, cross-validation, outcome-sensitivity, training-outcome, shot-type, component-level, and moderation analyses	This paper	Zenodo: https://doi.org/10.5281/zenodo.22828547
OpenPose	Cao et al.^10^	v1.7.0; BODY_25
YOLOv8s object detector	Ultralytics	https://github.com/ultralytics/ultralytics
Python	Python Software Foundation	v3.11; https://www.python.org/
PyTorch	PyTorch	v2.2; https://pytorch.org/
CUDA	NVIDIA	v12.1; https://developer.nvidia.com/cuda-toolkit
G∗Power	Faul et al.^31^	v3.1; https://www.psychologie.hhu.de/en/arbeitsgruppen/allgemeine-psychologie-und-arbeitspsychologie/gpower

**Other**		

Sony α6400 camera	Sony	Model α6400
Shimmer3 inertial measurement unit	Shimmer	Shimmer3 IMU
Galaxy Tab S9 tablet	Samsung	Galaxy Tab S9
NVIDIA RTX 4090 graphics processing unit	NVIDIA	RTX 4090; 24 GB
Basketball shooting rubric and locked feedback-cue library	This paper	Table S1
MSEEM model architecture and optimization settings	This paper	Tables S1, S2, and S3; STAR Methods

### Experimental model and study participant details

#### Research design

The study combined multimodal machine-learning development and validation with a randomized parallel-group basketball training experiment. Baseline assessment was followed by six supervised training sessions, an immediate post-test, and a delayed retention assessment. Participants were assigned to either MSEEM-based feedback or standard coaching feedback after completion of baseline assessment, and rubric-based post-test and retention scores were evaluated by assessors masked to group assignment. Recruitment was conducted from 12 January to 6 February 2026, baseline testing from 9 to 13 February, and the six training sessions from 16 February to 6 March at two sessions per week separated by 48–72 h. Post-testing was completed within 48 h of the final training session, and retention was assessed 14 days later on 21–22 March 2026. The institutional ethics committee of Anhui Traffic Vocational and Technical College approved the study protocol, including participant-consent procedures and collection of video and wearable-sensor data, on 8 January 2026 (BBST/2026/017). The experimental protocol, model-evaluation procedures, and training outcomes were defined before group assignment.

Participant age and sex were recorded at baseline; both male and female participants were included, and their baseline distribution is reported in Table 3.

#### Participants

##### Recruitment

Active basketball players were recruited through coach-distributed invitations to university teams, community clubs, and local training academies, supplemented by poster notices and briefing sessions at the study court. Sample size was determined *a priori* using G∗Power 3.1 for the primary between-group comparison of training-related change. A two-sided significance level of 0.05, statistical power of 0.80, and a standardized effect size of d = 0.48 indicated a minimum analyzable sample of approximately 140 participants.^31^ Allowing for approximately 10% attrition increased the recruitment target to 156 participants, consistent with the assumed medium effect magnitude.^32^ A total of 382 expressions of interest were received, of which 24 duplicate records were removed, leaving 358 individuals for screening (Figure S2). Of these, 66 did not meet the minimum basketball-experience criterion, 41 did not satisfy the participation-readiness requirements, and 52 reported activity restrictions that prevented unrestricted shooting participation, leaving 199 eligible individuals. Thirty-five did not attend the scheduled baseline assessment, resulting in 164 participants completing baseline testing. Eight withdrew before group assignment, leaving 156 participants who were allocated equally to the MSEEM-feedback condition (*n* = 78) and standard coaching condition (*n* = 78). All 156 participants completed the six training sessions, immediate post-test, and delayed retention assessment, with no post-assignment exclusions or missing primary-outcome observations.

#### Eligibility criteria

The inclusion criteria were (i) age 18–30 years, verified using national identification; (ii) at least 24 months of organized basketball experience, confirmed through team-roster records or coach verification; (iii) regular moderate-or-higher habitual physical activity assessed using the International Physical Activity Questionnaire–Short Form (IPAQ-SF)^33^; (iv) an IPAQ-SF activity level of at least 600 metabolic equivalent task-minutes per week according to the standard scoring protocol^34^; and (v) readiness to perform vigorous basketball activity assessed using the Physical Activity Readiness Questionnaire for Everyone (PAR-Q+).^35^ Positive PAR-Q+ responses required documented clearance for unrestricted participation before baseline assessment, following the extended screening procedure described by D. E. R. Warburton et al.^36^ The exclusion criteria were (i) a current physical complaint classified using the Oslo Sports Trauma Research Center Questionnaire on Health Problems as causing a moderate or severe reduction in training volume or sports performance, or preventing full participation in training or competition^37^; (ii) an acute lower-limb injury during the preceding six weeks that restricted normal basketball shooting practice; (iii) inability to complete the prescribed shooting volume without activity restriction; and (iv) inability to participate in all measurement modalities because of inability to wear the IMU sensors or non-consent to synchronized video recording.

#### Stratification and bias

Participants were assigned in a 1:1 ratio to the MSEEM-feedback or standard coaching condition using stratified block randomization. Stratification was based on baseline rubric-score tertile (lower, middle, or upper) and playing level (recreational club or competitive team), producing six allocation strata. Within each stratum, a computer-generated sequence used randomly varying permuted block sizes of four and six to maintain group balance while limiting predictability of assignment. The allocation sequence was generated before completion of baseline assessment and remained inaccessible to personnel conducting eligibility screening and baseline scoring until both procedures had been completed. Group assignment was then released sequentially for each eligible participant according to the corresponding stratum. This procedure maintained balanced assignment across the principal baseline performance and playing-level characteristics and followed established parallel-group randomization procedures.^38^ To reduce selection bias, recruitment scripts and screening procedures were standardized across sites. Procedural variation was reduced by standardizing shot volume, rest intervals, feedback timing and duration, court layout, lighting, camera positioning, and sensor-fusion procedures across groups and sessions. Baseline-score tertile and playing level were retained as prespecified covariates in the primary inferential models of training efficiency and retention.

### Method details

#### MSEEM implementation and data acquisition

##### Modalities and instrumentation setup

The experiment adopted MSEEM using RGB video, markerless pose keypoints, IMU signals, ball-trajectory features, and shot outcomes. Two Sony α6400 cameras recorded 1920 × 1080-pixel video at 120 frames/s with a 1/1000-s shutter. The frontal and sagittal cameras were mounted 2.4 m above the floor and approximately 6.0 m from the center of the shooting volume, with their optical axes intersecting the expected release region. OpenPose v1.7.0 using the BODY_25 configuration generated 2D keypoints independently in each view. Keypoints with confidence <0.50 were treated as missing; gaps of ≤100 ms were reconstructed by cubic interpolation, whereas longer gaps were retained as missing and contributed to the block-level quality criterion. Camera intrinsics and lens distortion were estimated before each acquisition day using a 9 × 6 checkerboard with 30-mm squares and at least 20 calibration views per camera. Calibration was accepted when mean reprojection error was ≤0.50 pixels. A court-fixed coordinate system placed the origin at the floor projection of the rim center, with the x-axis mediolateral, y-axis directed toward the shooting area, and z-axis vertical; the rim plane was fixed at z = 3.05 m. Corresponding keypoints from the two calibrated views were triangulated to obtain 3D joint coordinates, and reconstructed points with reprojection error >15 pixels were rejected.

Ball centers were detected in each synchronized view using a fine-tuned YOLOv8s detector with a confidence threshold of 0.40 and were triangulated using the same calibrated camera matrices. Gaps of no more than three video frames were interpolated before trajectory smoothing. Release angle was calculated from the initial 3D velocity vector over the first 120 ms after release, apex height from the maximum reconstructed z-coordinate, and entry angle from the descending 3D velocity vector at intersection with the 3.05-m rim plane. Two Shimmer3 IMU units were secured to the dorsal shooting hand and mid-forearm using non-slip elastic straps, with the longitudinal sensor axis aligned to each segment. Acceleration and angular velocity were sampled at 200 Hz using ranges of ±16 g and ±2000°/s. A 30-s stationary calibration preceded each session, and orientation drift assessed over a subsequent 60-s stationary interval averaged 0.38°/min and did not exceed 0.71°/min. Cross-modal synchronization used a simultaneous LED event and mechanical tap as synchronization events; residual alignment error across sessions had a median of 3.2 ms, an interquartile range of 2.5–4.1 ms, and a maximum of 8.6 ms, satisfying the predefined ≤10-ms tolerance. Markerless reconstruction was independently checked on a validation subset of 20 participants performing 10 representative shots each (200 shots) while synchronized reference trajectories were collected using a 10-camera Vicon system at 200 Hz. After temporal and coordinate alignment, the markerless pipeline produced a 3D wrist-position root mean square error of 21.4 mm, with mean absolute angular errors of 3.6° for elbow flexion, 4.1° for shoulder flexion, and 3.0° for trunk inclination. Agreement with the reference trajectories ranged from ICC = 0.90 to 0.95 across these measures. Ball-trajectory validation produced mean absolute differences of 2.2° for release angle, 0.05 m for apex height, and 2.6° for entry angle. These validation trials were excluded from model training and evaluation (Figure S1).

#### Shot task protocol and session schedule

A fixed session structure was used to provide comparable exposure and workload across participants and conditions while maintaining a consistent distribution of shot types. Each session comprised 12 blocks of 10 shots, yielding 120 shots in total, with each of the four shot types, free throw, catch-and-shoot, off-dribble, and lightly contested, performed in three blocks. The lightly contested condition used a standardized passive defender positioned 0.75 m in front of the shooter with one hand raised and no physical contact, providing controlled proximity interference without restricting the shooting motion. Rest intervals of 60 s were imposed between blocks and 180 s between shot-type transitions, and total session duration was recorded to support calculation of score improvement per session-minute. Catch-and-shoot trials began from a designated 4.6 m mark and were initiated by a standardized chest pass delivered by an assistant at a fixed cadence. Off-dribble trials began from a 2.0 m starting mark and used a single-dribble approach to standardize movement toward the basket. Blocks affected by interruptions, sensor displacement, camera obstruction, or failure to meet predefined data-quality criteria, defined as >2% missing video frames or >1 s of IMU dropout, were repeated after the acquisition problem had been corrected.

Each participant therefore completed 30 attempts per shooting technique and 120 attempts per session. The baseline model-development dataset contained 18,720 scheduled shots from 156 participants, comprising 4,680 free throws, 4,680 catch-and-shoot attempts, 4,680 off-dribble attempts, and 4,680 lightly contested attempts. Across the six training sessions, each participant completed 720 scheduled shots; each study condition therefore contributed 56,160 training shots from 78 participants, corresponding to 14,040 attempts per shooting technique per condition. Immediate post-test and retention assessments each contributed a further 18,720 scheduled attempts under the same 30-shot-per-technique distribution. For baseline generalization testing, the three 10-shot repetitions of each shooting technique were acquired under three prespecified recording environments presented in counterbalanced order. Environment E1 used the nominal camera geometry and court illumination of approximately 550 ± 50 lx. Environment E2 retained the same camera geometry with illumination reduced to approximately 350 ± 50 lx. Environment E3 retained nominal illumination while the sagittal camera was displaced 0.50 m laterally and rotated 10° toward the shooting volume. Each participant therefore contributed 40 baseline shots per environment, yielding 6,240 shots per environment and 1,560 shots for each technique–environment combination. The six training sessions, post-test, and retention assessment used the standardized E1 configuration; environmental perturbations were restricted to baseline generalization testing.

#### Reference rubric labels and rater reliability

The reference rubric was finalized before model development and independently of the analytical dataset. It generated a total shooting-skill score of 0–100 from four equally weighted components: alignment, timing, release mechanics, and stability, each scored from 0 to 25. Component definitions and score-band anchors were based on observable shooting technique and were fixed before model-generated features or predictions were examined. Five score bands were applied within each component: 0–5, substantial technical inconsistency; 6–10, limited control; 11–15, adequate but variable execution; 16–20, proficient and generally consistent execution; and 21–25, highly consistent execution. Shot outcome was excluded from all rubric criteria. Two certified basketball coaches completed two 90-min calibration sessions using 30 pilot blocks excluded from model development and evaluation. During formal scoring, the coaches reviewed synchronized video without access to MSEEM scores, derived kinematic variables, study condition, shot-outcome labels, or the other coach’s ratings. Ratings were completed independently and locked before model fitting. The mean of the two ratings formed the reference label when disagreement remained within 10 points for the total score and 5 points for an individual component; discrepancies exceeding either threshold triggered joint review of the original synchronized video before the final reference score was sealed. Inter-rater reliability was quantified using a two-way random-effects, absolute-agreement model.^39^^,^^40^ Single-rater reliability was ICC (2,1) = 0.79 (95% CI 0.75–0.83), while reliability of the two-rater mean used as the reference score was ICC (2,2) = 0.88 (95% CI 0.85–0.91). ICC represented agreement between raters; independence of the reference standard was established through prior rubric specification, masked scoring, and exclusion of model-derived information from label construction (Table S1).

#### Model development, baselines, and feedback delivery

Model development and evaluation were conducted using baseline-session data collected before group assignment and before exposure to any feedback. The baseline dataset was evaluated using five-fold cross-validation to estimate the generalization of rubric-score prediction and to compare MSEEM with modality ablations and external comparator models. After model selection, a single deployment model was trained using all baseline-session shots and the locked coach-derived rubric labels. The resulting model weights were then frozen before the first training session, and no post-test or retention labels were used for model fitting, tuning, or updating. Generalization analyses used separate splitting procedures for player, environment, and session transfer. Cross-player evaluation used five outer folds grouped at participant level, containing 32, 31, 31, 31, and 31 held-out participants, respectively; every baseline shot from a participant remained within the same outer fold. For the fold containing 32 test participants, the remaining 124 participants were divided into 99 training and 25 validation participants; folds containing 31 test participants used 100 training, 25 validation, and 31 test participants. The resulting held-out sets contained 3,720–3,840 baseline shots per fold. Feature scaling, missing-value handling, hyperparameter selection, and early-stopping decisions were estimated exclusively from training and inner-validation observations and were frozen before outer-fold testing.

Cross-environment evaluation used leave-one-environment-out testing across E1, E2, and E3. Two environments contributed 12,480 baseline shots for model development and the third contributed 6,240 held-out shots, with the held-out environment rotated across three analyses. Participants could occur in both the development and held-out environmental sets because this analysis evaluated environment transfer specifically; participant-independent generalization was evaluated separately by the cross-player splits. Cross-session evaluation used the deployment model fitted to baseline observations and frozen before the first training session. Prediction was subsequently evaluated across sessions 1–6 without refitting, recalibration, normalization updates, or session-specific hyperparameter selection. Post-test and retention observations were excluded from model development and tuning (Table S2).

#### Preprocessing and feature construction

Each shot was segmented around the release event to standardize multimodal alignment across variations in shooting tempo. Release time was defined as the first frame in which ball-to-wrist separation exceeded 0.25 m for at least five consecutive frames and estimated ball speed exceeded 1.0 m/s. This estimate was then refined using the nearest peak in wrist angular velocity within ±120 ms. All modality streams were re-timestamped to a common 200 Hz timebase, with RGB video and markerless pose features interpolated using cubic interpolation and IMU signals retained at their native sampling rate. Joint-angle time series were smoothed using a zero-lag fourth-order Butterworth filter with a 10 Hz cutoff, while IMU acceleration and angular-velocity signals were smoothed using a zero-lag fourth-order Butterworth filter with a 20 Hz cutoff. A fixed analysis window from −600 ms to +800 ms relative to release was applied to each shot, and block-level consistency was quantified as within-block variability across the 10 shots (Table 1). Label independence was maintained by using coach-derived rubric scores as the reference labels, while shot outcome remained an input feature and was excluded from both label construction and rater scoring.

#### Model training, external comparators, and ablations

The full MSEEM architecture used a late-encoder gated-fusion design with parallel modality-specific encoders and a multi-task scoring head (Table 2). Video-derived contextual features and ball-trajectory features were embedded using separate two-layer multilayer perceptrons with 128-dimensional outputs. Pose and IMU sequences were processed using separate temporal transformer encoders, each with four attention heads, two layers, and a 128-dimensional model size. Shot outcome was represented as a learned 16-dimensional token. The encoded modality streams were combined through a gated attention layer that learned modality-specific weights for each shot window, followed by a 256-unit fusion layer. The output stage comprised one regression head for the total shooting score from 0 to 100 and four component heads for alignment, timing, release mechanics, and stability, each ranging from 0 to 25. These outputs were trained jointly using a weighted loss to preserve component-level interpretability. All ablation models were trained and evaluated using baseline-session data under identical data splits. After model selection, the final MSEEM deployment model was trained once on the complete baseline dataset and frozen before feedback delivery. Ablations removed one modality at a time, including RGB video, pose, IMU, ball trajectory, or shot outcome, replaced gated fusion with simple concatenation to assess the contribution of each design element under the same evaluation protocol.

Outcome-stream dependence was examined after each outer-fold model fit using a counterfactual outcome-flip analysis. For every held-out shot, the binary make/miss input was reversed while all other modality inputs remained unchanged, and the resulting change in predicted total score was recorded. Mean gated-attention weights were also extracted for each modality across held-out observations. These analyses were diagnostic and did not contribute to model fitting, hyperparameter selection, or deployment-model selection. External comparators were implemented from published architectures and adapted to predict the rubric score by replacing their original classification heads with regression heads. All comparator models were trained using the same baseline-session cross-validation folds. For video-only comparison, SlowFast was implemented as a two-pathway convolutional video model.^41^ TimeSformer provided an attention-based video-transformer comparator.^42^ Video Swin Transformer represented a windowed spatiotemporal transformer with locality-aware processing.^43^ For pose-only comparison, OpenPose keypoints were processed using a Spatial-Temporal Graph Convolutional Network to preserve structured dependencies among joints.^44^ For IMU-only comparison, DeepConvLSTM was used as a convolutional-recurrent baseline.^45^ A transformer-based inertial model provided the attention-based temporal comparator.^46^ All comparator training was restricted to baseline-session data to prevent information from the training, post-test, or retention phases from influencing model construction.

Model topology, input modalities, component heads, analysis window, and feedback-selection rule were fixed before comparative evaluation. Hyperparameter selection was conducted only within the training portion of each outer split using an equal budget of 20 candidate configurations per model. Candidate values comprised learning rates of 1 × 10^-4^, 3 × 10^-4^, and 1 × 10^-3^; dropout of 0.10, 0.20, and 0.30; encoder depths of one, two, and three layers; two, four, or eight attention heads where architecture-compatible; and Huber delta values of 0.5, 1.0, and 2.0. The configuration with the lowest inner-validation error was selected and fixed before outer-fold evaluation. External architectures retained their native backbone capacities; comparison fairness was therefore maintained through identical data partitions, preprocessing, candidate-search budget, optimization opportunities, early-stopping criteria, and test observations. Training was implemented using Python 3.11, PyTorch 2.2, and CUDA 12.1 on one NVIDIA RTX 4090 GPU with 24 GB memory. Trainable parameter counts after adaptation of the task-specific output heads were approximately 0.74 million for MSEEM, 34.5 million for SlowFast, 121.4 million for TimeSformer, 28.2 million for Video Swin Transformer, 3.10 million for ST-GCN, 1.17 million for DeepConvLSTM, 1.46 million for the IMU Transformer, and 0.04 million for the outcome-only model. MSEEM ablations contained approximately 0.72 million parameters without RGB, 0.47 million without pose, 0.47 million without IMU, 0.72 million without ball trajectory, 0.74 million without outcome, 0.69 million with concatenation fusion. Median final-fit time per outer fold was 22 min for MSEEM, 41 min for SlowFast, 67 min for TimeSformer, 52 min for Video Swin Transformer, 12 min for ST-GCN, 8 min for DeepConvLSTM, 11 min for the IMU Transformer, and less than 2 min for the outcome-only model. Hyperparameter search and final cross-validation required approximately 186 GPU-hours in total (Table S3). An outcome-only comparator used the binary make/miss indicator as its sole input for rubric-score prediction.

#### Feedback implementation and dosage control

MSEEM feedback was delivered on a tablet computer (Galaxy Tab S9, Samsung, South Korea) within 5 s after each 10-shot block. The pipeline performed block closure, automatic data-integrity checks with missing frames ≤2% and IMU dropout ≤1 s, and inference using the pre-trained, frozen deployment model. Prompt selection followed a fixed rule that identified the two largest component deficits relative to the 25-point component maximum and returned the corresponding cues from a locked cue library. Model parameters were not updated during or between training sessions, or after post-test and retention assessment, and study outcomes were derived exclusively from blinded coach rubric scores; model outputs were not used as study outcomes. Control feedback was delivered at the same block cadence and with comparable frequency and duration, but without numerical scores, component subscores, rankings, or tablet-based displays. Adherence was recorded as the number of completed blocks and feedback prompts delivered per session in both conditions. Training-related adverse events that interrupted a block, required cessation of participation, or prevented completion of the scheduled session were recorded throughout the six-session training period.

### Quantification and statistical analysis

Model performance for MSEEM, modality ablations, and external comparators was evaluated using five-fold cross-validation on baseline-session data under prespecified cross-player, cross-session, and cross-environment splits. Primary RQ1 endpoints were mean absolute error, $\text{MAE}=\frac{1}{N}\sum_{i=1}^{N}\left|y_{i}-{\hat{y}}_{i}\right|$, and Spearman rank correlation, $\rho_{s}=1-\frac{6\sum_{i=1}^{N}d_{i}^{2}}{N\left(N^{2}-1\right)}$, where $d_{i}=r\left(y_{i}\right)-r\left({\hat{y}}_{i}\right)$. Calibration was assessed using the linear calibration model $y_{i}=\alpha +\beta {\hat{y}}_{i}+\epsilon_{i}$, reporting intercept α and slope β. For RQ2, training efficiency was defined as score gain per session-minute across six sessions, modeled at the participant level from repeated rubric scores, and retention was defined as Δ_ret,i_=y_i,ret_-y_i,post_ on the same shot set under identical testing conditions.

For the primary computational analysis, MSEEM was compared with seven external comparators and six prespecified ablation models, producing 13 paired comparisons for each primary performance measure. Fold observations were retained as paired units, and corrected resampling was applied to within-fold differences without treating the five outer folds as independent replications. Holm adjustment was performed separately across the 13 mean-absolute-error comparisons and the 13 rank-agreement comparisons. Calibration intercept and slope were treated as secondary descriptive measures. Training efficiency and delayed retention were the prespecified training outcomes. Training efficiency was estimated using a participant-level mixed-effects model containing fixed effects for condition, cumulative practice time, condition × time, baseline rubric score, playing level, and baseline-score tertile. Participant-specific random intercepts and random slopes for cumulative practice time were fitted with an unstructured intercept–slope covariance matrix. Models were estimated using restricted maximum likelihood, with Satterthwaite approximation for fixed-effect degrees of freedom and 95% confidence intervals. The retention model retained the same baseline and stratification covariates and included post-test versus retention timepoint as the within-participant factor. Holm adjustment was applied across the two primary training contrasts. Shot-type and component-level analyses were classified as secondary and adjusted within their respective four-outcome families; moderation analyses were exploratory and interpreted using effect estimates and 95% confidence intervals. Standardized retention effects were calculated by dividing the adjusted between-condition difference by the pooled observed retention-score standard deviation. Retention-score standard deviations of 8.5 and 8.9 points in the two groups produced a pooled standard deviation of 8.70 points; consequently, the adjusted difference of 5.90 points corresponded to d = 0.68. Repeated-measures models used likelihood-based estimation under a missing-at-random assumption, with complete-case estimation retained as a sensitivity analysis where missing observations occurred.

## Supplemental information

Document S1. Figures S1–S5 and Tables S5–S7

Table S1. Complete shooting-technique rubric and locked feedback-cue library

Table S2. Independence structure and observation counts for model-development and generalization analyses

Table S3. Computational capacity and optimization budget for MSEEM, modality ablations, and external comparators

Table S4. Fold-level paired model-comparison results

## References

[1] Caruso M., Cimmino L., Narducci F., Pero C., Ronga G. (2025). Advancements in basketball action recognition: Datasets, methods, explainability, and synthetic data applications. Image Vis Comput..

[2] Nowak M., Skalik M., Więckowski J., Ciejpa R., Stolarczyk A., Oleksy Ł. (2025). Winners’ strategies: Comprehensive analysis and optimization of 2-point shots in 3x3 basketball using multi-criteria decision support analysis, on the example of two Olympic National Teams. PLoS One.

[3] Fang K.Q., Jiang H. (2025). A machine learning framework for evaluating basketball player skill development using video analysis. Egyptian Informatics Journal.

[4] Jia Y., Anida Abdullah N., Eliza H., Lu Q., Si D., Guo H., Wang W. (2025). A narrative review of deep learning applications in sports performance analysis: current practices, challenges, and future directions. BMC Sports Sci. Med. Rehabil..

[5] Zheng C., Zhou Y. (2025). Multi-modal IoT data fusion for real-time sports event analysis and decision support. Alex. Eng. J..

[6] Asadi A., Daneshfar A., Saeedpour-Parizi M.R., Aiken C.A., Smiley A. (2025). Detecting optimal gaze behavior of successful basketball free throwing via machine learning system. Hum. Mov. Sci..

[7] Zhang Z., Liu W., Zheng Y., Du L., Sun L. (2025). Learning spatio-temporal context for basketball action pose estimation with a multi-stream network. Sci. Rep..

[8] Deng H. (2025). Basketball free throw hit probability prediction system based on deep learning object detection and pose estimation. Systems and Soft Computing.

[9] Zhao B., Liu S. (2021). Basketball shooting technology based on acceleration sensor fusion motion capture technology. EURASIP J. Adv. Signal Process..

[10] Cao Z., Hidalgo G., Simon T., Wei S.E., Sheikh Y. (2021). OpenPose: Realtime Multi-Person 2D Pose Estimation Using Part Affinity Fields. IEEE Trans. Pattern Anal. Mach. Intell..

[11] Varcin F., Boocock M.G. (2026). The accuracy, validity and reliability of Theia3D markerless motion capture for studying the biomechanics of human movement: A systematic review. Artif. Intell. Med..

[12] Verhoeven F.M., Newell K.M. (2016). Coordination and control of posture and ball release in basketball free-throw shooting. Hum. Mov. Sci..

[13] Nakano N., Iino Y., Inaba Y., Fukashiro S., Yoshioka S. (2022). Utilizing hierarchical redundancy for accurate throwing movement. Hum. Mov. Sci..

[14] Cabarkapa D., Cabarkapa D.V., Miller J.D., Templin T.T., Frazer L.L., Nicolella D.P., Fry A.C. (2023). Biomechanical characteristics of proficient free-throw shooters-markerless motion capture analysis. Front. Sports Act. Living.

[15] Khobdeh S.B., Yamaghani M.R., Sareshkeh S.K. (2023). Basketball action recognition based on the combination of YOLO and a deep fuzzy LSTM network. J. Supercomput..

[16] Liu J., Liu X., Qu M., Lyu T. (2025). EITNet: An IoT-enhanced framework for real-time basketball action recognition. Alex. Eng. J..

[17] Huang R., Zhang B., Yao Z., Xie B., Guo J. (2025). DESNet: Real-time human pose estimation for sports applications combining IoT and deep learning. Alex. Eng. J..

[18] Chen Q., Liu Z., He D., Tang Y., Gao Y. (2026). A Machine Learning-Based Model for Comprehensive Assessment and Classification of Cross-Country Skiing Athletes. Res. Q. Exerc. Sport.

[19] Hsu W.-C., Lee C.-C., Lee Y.-H., Lin Y.-J., Hung Y.-C., Ho C.-C., Chang Y.-C. (2026). PoseShot: hybrid CNN–BiLSTM transformer model for free throw action recognition via pose analysis. Sci. Rep..

[20] Seino T., Saito N., Ogawa T., Asamizu S., Haseyama M. (2025). Expert Comment Generation Considering Sports Skill Level Using a Large Multimodal Model with Video and Spatial-Temporal Motion Features. Sensors (Basel).

[21] Wang J., Zuo L., Cordente Martínez C. (2024). Basketball technique action recognition using 3D convolutional neural networks. Sci. Rep..

[22] Zhang L. (2024). Deep learning based fine-grained recognition technology for basketball movements. Systems and Soft Computing.

[23] Dong L., Hu T., Sun T., Li J., Ma S. (2025). RGB video and inertial sensing fusion method for human action recognition in human-robot collaborative manufacturing. J. Manuf. Syst..

[24] Xefteris V.-R., Contiero Syropoulou A., Pistola T., Kasnesis P., Poulios I., Tsanousa A., Symeonidis S., Diplaris S., Goulianas K., Chatzimisios P., Vrochidis S. (2025). Multimodal fusion of inertial sensors and single RGB camera data for 3D human pose estimation based on a hybrid LSTM-Random forest fusion network. Internet Things.

[25] Khan S.U., Sultana M., Danish S., Gupta D., Alghamdi N.S., Woo S., Lee D.-G., Ahn S. (2025). Multimodal feature fusion for human activity recognition using human centric temporal transformer. Eng. Appl. Artif. Intell..

[26] Sha N. (2025). Basketball technical action recognition based on a combination of capsule neural network and augmented red panda optimizer. Egyptian Informatics Journal.

[27] Petancevski E.L., Inns J., Fransen J., Impellizzeri F.M. (2022). The effect of augmented feedback on the performance and learning of gross motor and sport-specific skills: A systematic review. Psychol. Sport Exerc..

[28] Ueyama Y., Harada M. (2024). Basketball free-throw training with augmented reality-based optimal shot trajectory for novice shooters. Sci. Rep..

[29] He X., Wei L. (2025). Real-time feedback enhances motor learning and motivation in youth team sports through augmented reality tools [Original Research]. Front. Psychol..

[30] Ma J., Ma L., Qi S., Zhang B., Ruan W. (2025). A practical study of artificial intelligence-based real-time feedback in online physical education teaching. Smart Learn. Environ..

[31] Faul F., Erdfelder E., Lang A.-G., Buchner A. (2007). G∗Power 3: A flexible statistical power analysis program for the social, behavioral, and biomedical sciences. Behav. Res. Methods.

[32] Cohen J. (1992). Statistical Power Analysis. Curr. Dir. Psychol. Sci..

[33] Craig C.L., Marshall A.L., SjÖStrÖM M., Bauman A.E., Booth M.L., Ainsworth B.E., Pratt M., Ekelund U.L.F., Yngve A., Sallis J.F., Oja P. (2003). International Physical Activity Questionnaire: 12-Country Reliability and Validity. Med. Sci. Sports Exerc..

[34] Maddison R., Ni Mhurchu C., Jiang Y., Vander Hoorn S., Rodgers A., Lawes C.M., Rush E. (2007). International Physical Activity Questionnaire (IPAQ) and New Zealand Physical Activity Questionnaire (NZPAQ): a doubly labelled water validation. Int. J. Behav. Nutr. Phys. Act..

[35] Warburton D.E.R., Jamnik V.K., Bredin S.S.D., McKenzie D.C., Stone J., Shephard R.J., Gledhill N. (2011). Evidence-based risk assessment and recommendations for physical activity clearance: an introduction. Appl. Physiol. Nutr. Metab..

[36] Warburton D.E.R., Jamnik V.K., Bredin S.S.D., Gledhill N. (2011). The Physical Activity Readiness Questionnaire for Everyone (PAR-Q+) and Electronic Physical Activity Readiness Medical Examination (ePARmed-X+). The Health and Fitness Journal of Canada.

[37] Clarsen B., Rønsen O., Myklebust G., Flørenes T.W., Bahr R. (2014). The Oslo Sports Trauma Research Center questionnaire on health problems: a new approach to prospective monitoring of illness and injury in elite athletes. Br. J. Sports Med..

[38] Schulz K.F., Altman D.G., Moher D., CONSORT Group (2010). CONSORT 2010 Statement: updated guidelines for reporting parallel group randomised trials. BMJ.

[39] Koo T.K., Li M.Y. (2016). A Guideline of Selecting and Reporting Intraclass Correlation Coefficients for Reliability Research. J. Chiropr. Med..

[40] Shrout P.E., Fleiss J.L. (1979). Intraclass correlations: uses in assessing rater reliability. Psychol. Bull..

[41] Feichtenhofer C., Fan H., Malik J., He K. (2019). 2019 IEEE/CVF International Conference on Computer Vision (ICCV).

[42] Bertasius G., Wang H., Torresani L. (2021). Is Space-Time Attention All You Need for Video Understanding?. Proceedings of the 38th International Conference on Machine Learning.

[43] Liu Z., Ning J., Cao Y., Wei Y., Zhang Z., Lin S., Hu H. (2022). 2022 IEEE/CVF Conference on Computer Vision and Pattern Recognition (CVPR).

[44] Yan S., Xiong Y., Lin D. (2018). Spatial Temporal Graph Convolutional Networks for Skeleton-Based Action Recognition. Proc. AAAI Conf. Artif. Intell..

[45] Ordóñez F.J., Roggen D. (2016). Deep Convolutional and LSTM Recurrent Neural Networks for Multimodal Wearable Activity Recognition. Sensors (Basel).

[46] Shavit Y., Klein I. (2021). Boosting Inertial-Based Human Activity Recognition With Transformers. IEEE Access.

