# Supplementary material for: Automated evaluation of basketball skills using multimodal data fusion for improving shooting training efficiency
Source: iScience. 2026 Oct 15;29(11):117915. doi: 10.1016/j.isci.2026.117915 (PMC13694890; doi:10.1016/j.isci.2026.117915)
Supplement: Supplementary file 1 [file mmc1.pdf]

iScience, Volume *29*

Supplemental information

Automated evaluation of basketball skills using multimodal data fusion for improving shooting training efficiency

Puzi Sun

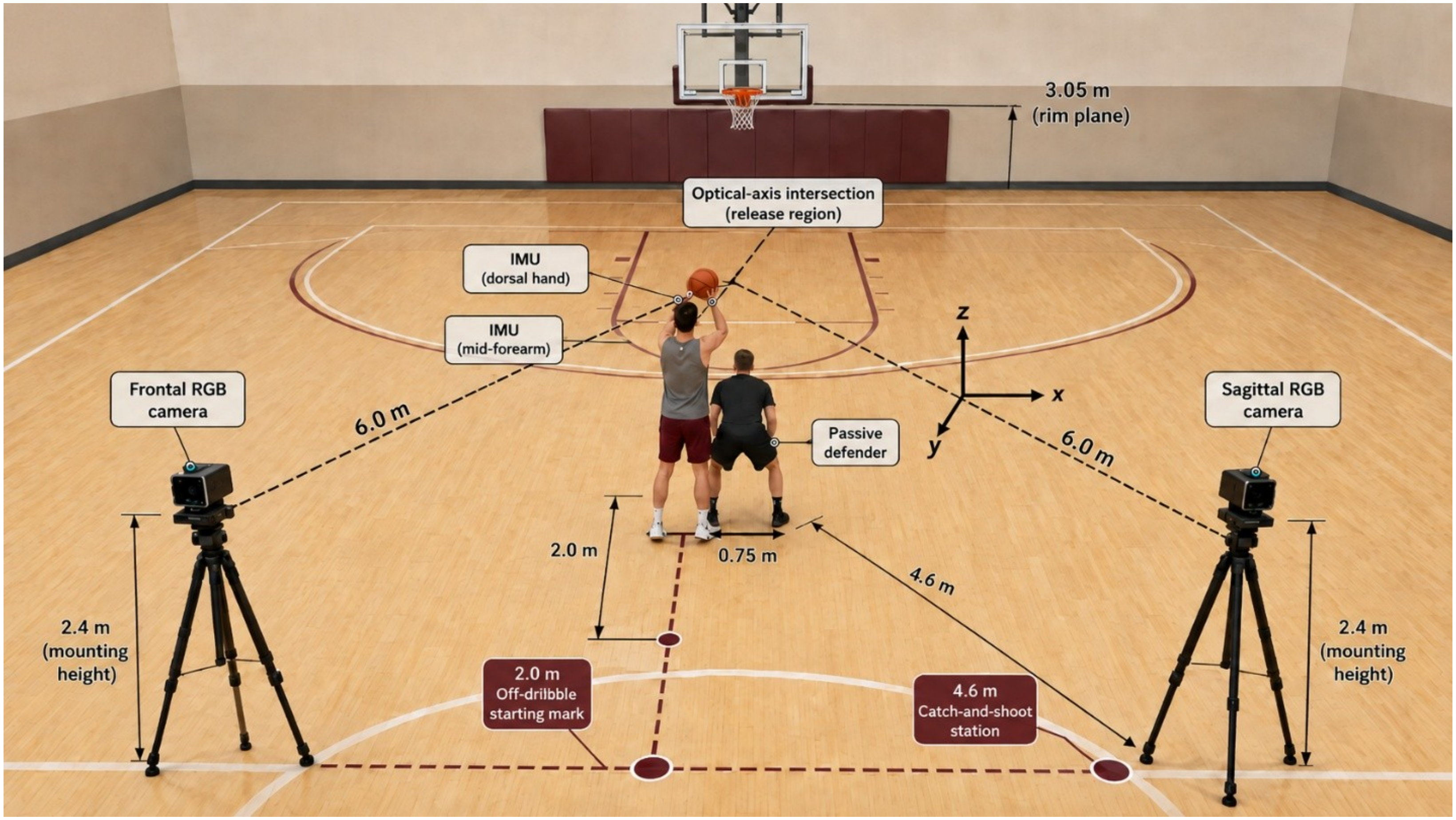

Figure S1. Basketball shooting acquisition geometry and sensor placement are shown for the standardized recording setup.

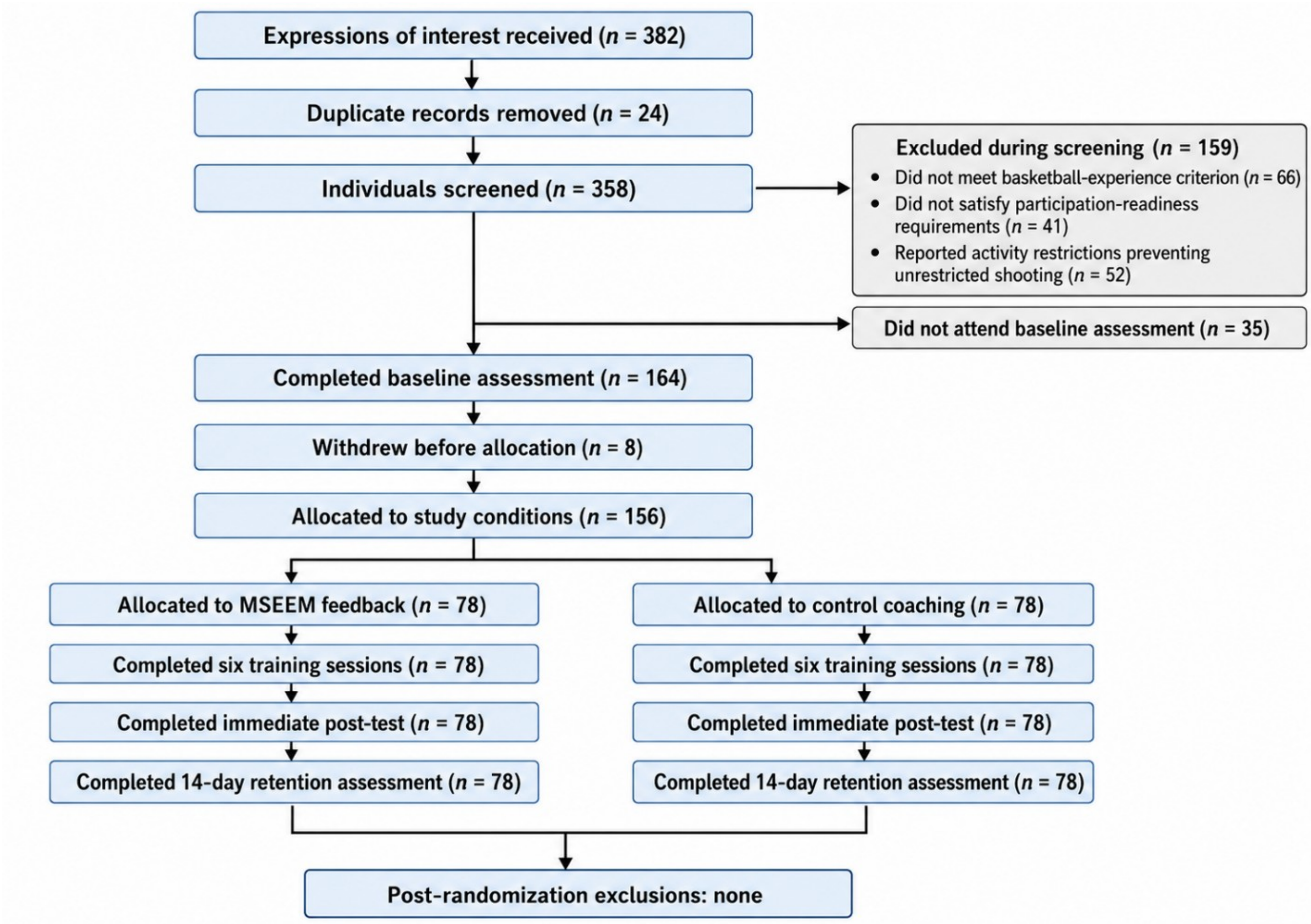

Figure S2. Participant flow is shown from screening through the 14-day retention assessment.

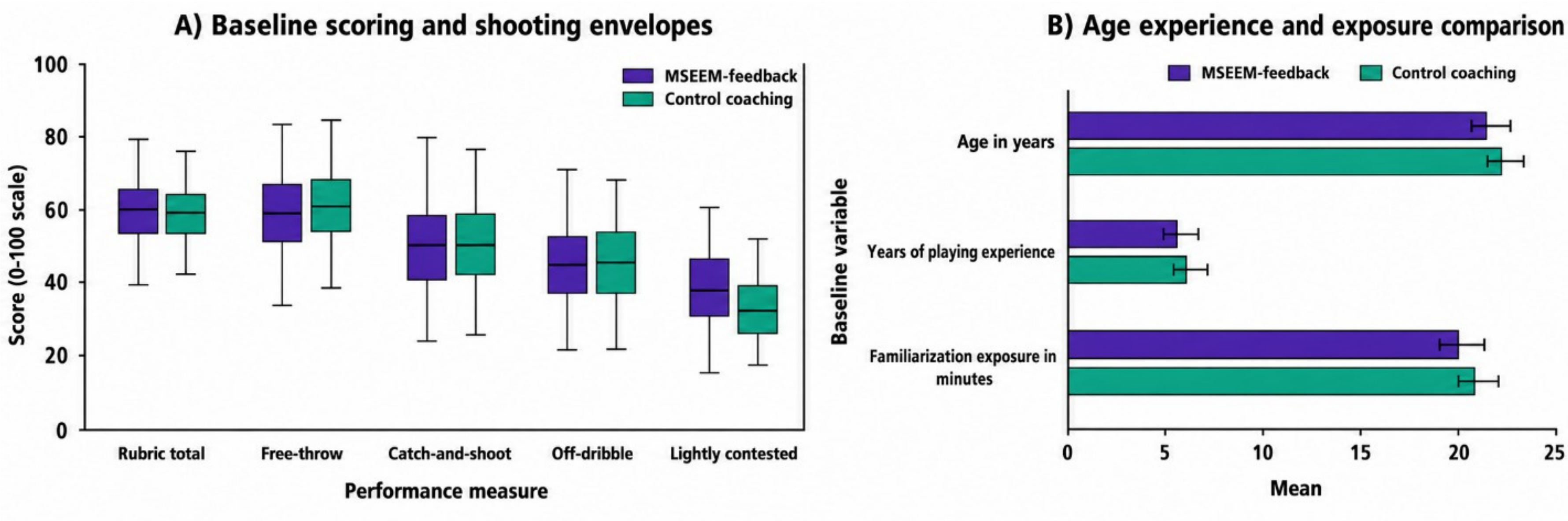

Figure S3. Baseline scoring distributions and participant characteristics are shown by study condition. (A) Baseline rubric total and shot-type distributions for MSEEM-feedback and control coaching. Boxes show the interquartile range with median lines, and whiskers show the displayed minimum and maximum values. (B) Baseline participant profile comparison for age, playing experience, and familiarization exposure. Bars show means and horizontal error bars represent 95% confidence intervals.

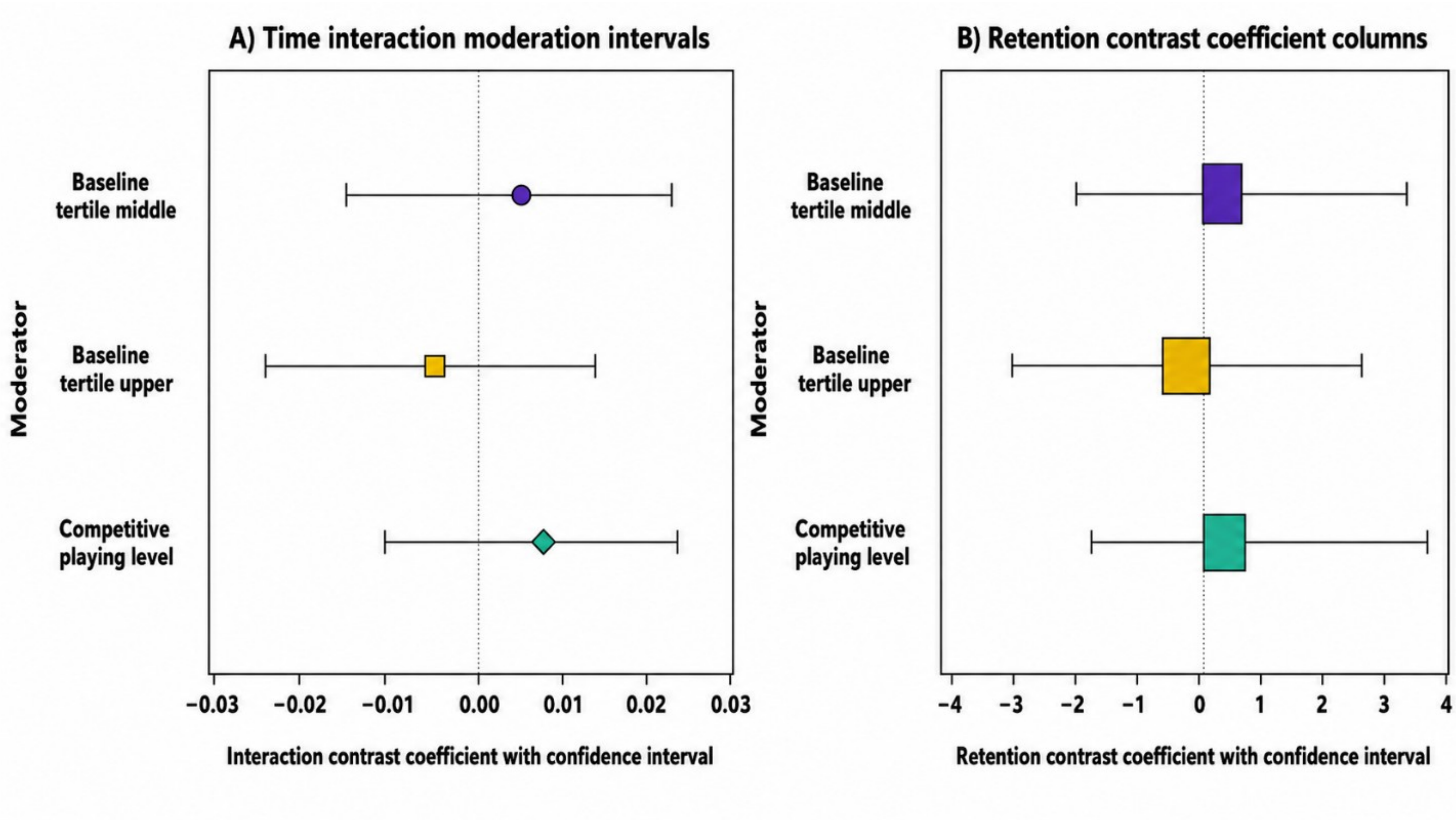

Figure S4. Moderator estimates for training and retention effects are shown across baseline tertiles and playing levels. (A) Moderation interval estimates for baseline tertile and playing-level interactions with the MSEEM-feedback × time effect. (B) Moderation-linked retention contrast estimates for baseline tertiles and competitive playing level. Markers and horizontal bars represent adjusted coefficients and 95% confidence intervals.

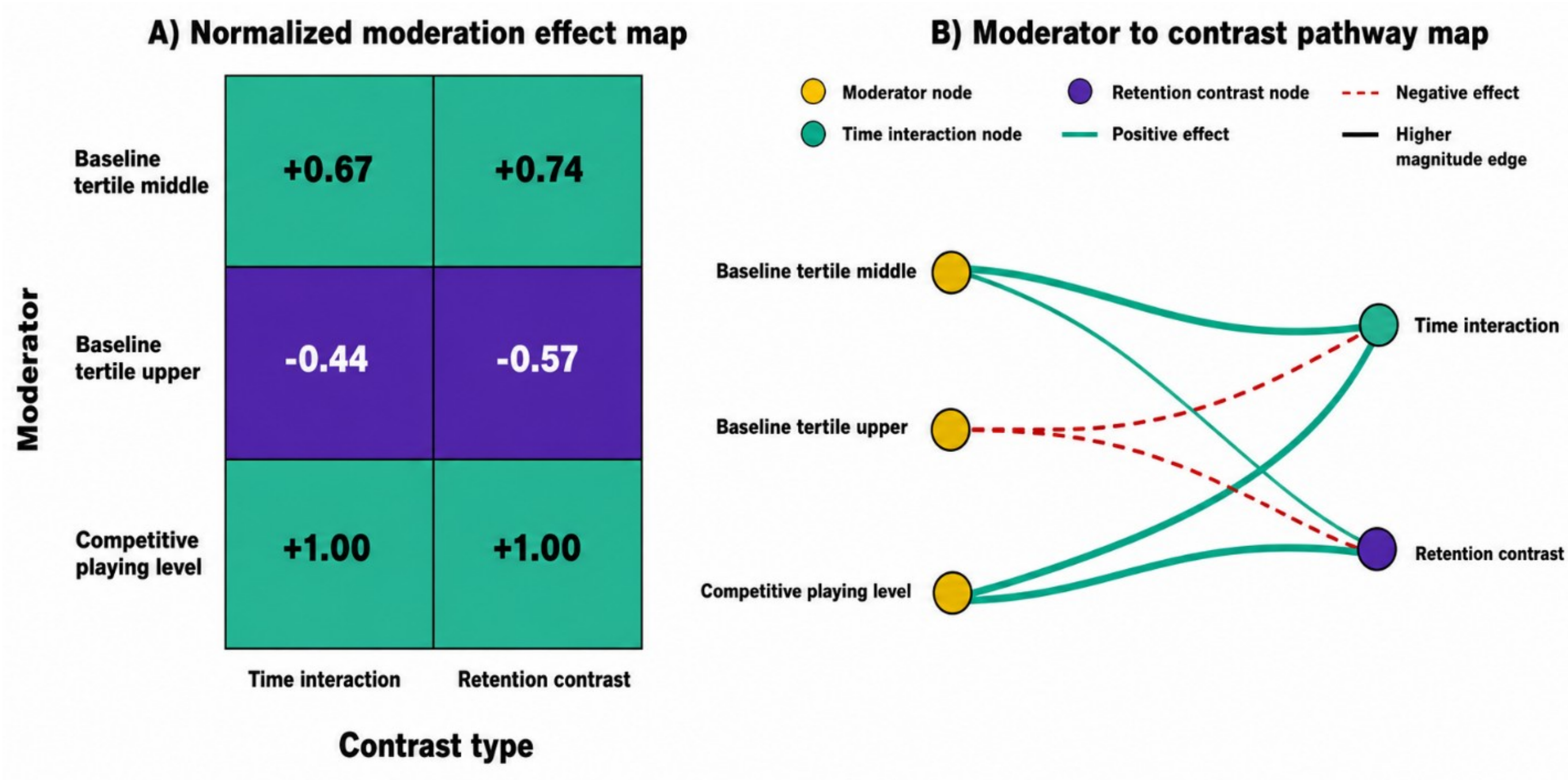

Figure S5. Exploratory moderator directions are summarized across time-interaction and retention contrasts. (A) Normalized moderation effect map for baseline tertile and playing-level moderator directions. (B) Moderator-to-contrast pathway map showing signed connections from moderators to time-interaction and retention nodes, with edge styling reflecting effect direction and relative magnitude.

Table S5. Component-score prediction performance

Model	Alignment MAE	Alignment rho	Alignment slope	Timing MAE	Timing rho	Timing slope	Release MAE	Release rho	Release slope	Stability MAE	Stability rho	Stability slope
MSEEM	1.12 ± 0.07	0.812 ± 0.022	0.99 ± 0.05	1.18 ± 0.08	0.804 ± 0.024	0.97 ± 0.06	1.25 ± 0.09	0.793 ± 0.026	0.96 ± 0.06	1.10 ± 0.07	0.820 ± 0.021	0.98 ± 0.05
Ablation without RGB video	1.26 ± 0.08	0.776 ± 0.025	0.94 ± 0.06	1.30 ± 0.09	0.770 ± 0.027	0.92 ± 0.07	1.40 ± 0.10	0.754 ± 0.029	0.91 ± 0.07	1.24 ± 0.08	0.784 ± 0.024	0.93 ± 0.06
Ablation without pose	1.48 ± 0.10	0.721 ± 0.030	0.90 ± 0.07	1.32 ± 0.09	0.762 ± 0.028	0.91 ± 0.07	1.58 ± 0.11	0.710 ± 0.032	0.88 ± 0.08	1.46 ± 0.10	0.736 ± 0.028	0.90 ± 0.07
Ablation without IMU	1.20 ± 0.08	0.795 ± 0.023	0.97 ± 0.06	1.46 ± 0.10	0.711 ± 0.031	0.88 ± 0.08	1.33 ± 0.10	0.776 ± 0.027	0.93 ± 0.07	1.18 ± 0.08	0.801 ± 0.022	0.96 ± 0.06
Ablation without ball trajectory	1.22 ± 0.08	0.789 ± 0.024	0.96 ± 0.06	1.24 ± 0.08	0.792 ± 0.023	0.95 ± 0.06	1.52 ± 0.11	0.724 ± 0.031	0.89 ± 0.08	1.16 ± 0.08	0.806 ± 0.022	0.96 ± 0.06
Comparator Video Swin Transformer	1.62 ± 0.12	0.694 ± 0.034	0.87 ± 0.08	1.66 ± 0.12	0.681 ± 0.035	0.86 ± 0.09	1.70 ± 0.13	0.668 ± 0.036	0.85 ± 0.09	1.58 ± 0.12	0.703 ± 0.033	0.87 ± 0.08
Comparator ST-GCN	1.70 ± 0.13	0.672 ± 0.036	0.85 ± 0.09	1.72 ± 0.13	0.664 ± 0.037	0.84 ± 0.09	1.78 ± 0.14	0.652 ± 0.038	0.83 ± 0.10	1.64 ± 0.13	0.690 ± 0.034	0.86 ± 0.09
Comparator IMU Transformer	1.58 ± 0.11	0.705 ± 0.033	0.88 ± 0.08	1.54 ± 0.11	0.704 ± 0.033	0.87 ± 0.08	1.62 ± 0.12	0.688 ± 0.035	0.86 ± 0.09	1.56 ± 0.11	0.707 ± 0.032	0.88 ± 0.08
Comparator outcome-only	1.88 ± 0.15	0.610 ± 0.045	0.78 ± 0.10	1.96 ± 0.16	0.598 ± 0.046	0.76 ± 0.10	2.04 ± 0.17	0.586 ± 0.047	0.74 ± 0.11	1.90 ± 0.15	0.606 ± 0.045	0.77 ± 0.10

Table S6. Shot-type training effects

Shot type	MSEEM-feedback × Time B ± SE	95% CI	t	Holm-adjusted p	Retention contrast B ± SE	95% CI	t	Holm-adjusted p
Free throw	0.041 ± 0.014	0.013 to 0.069	2.930	0.009	5.10 ± 1.92	1.32 to 8.88	2.660	0.018
Catch-and-shoot	0.039 ± 0.013	0.013 to 0.065	3.000	0.009	6.20 ± 2.05	2.16 to 10.24	3.020	0.009
Off-dribble	0.031 ± 0.013	0.005 to 0.057	2.390	0.017	4.10 ± 1.90	0.35 to 7.85	2.160	0.032
Lightly contested	0.048 ± 0.015	0.019 to 0.077	3.260	0.004	7.30 ± 2.18	3.00 to 11.60	3.350	0.004

Note. B represents the adjusted regression coefficient, SE the standard error, and CI the confidence interval. Holm adjustment was applied separately across the four shot-type comparisons for the MSEEM-feedback × Time effects and across the four retention contrasts.

Table S7. Component-level training effects

Component	MSEEM-feedback × Time B ± SE	95% CI	t	Holm-adjusted p	Post-test contrast B ± SE	95% CI	t	Holm-adjusted p	Retention contrast B ± SE	95% CI	t	Holm-adjusted p
Alignment	0.012 ± 0.004	0.004 to 0.020	3.000	0.009	1.50 ± 0.52	0.47 to 2.53	2.880	0.015	1.90 ± 0.58	0.75 to 3.05	3.280	0.004
Timing	0.010 ± 0.004	0.002 to 0.018	2.520	0.024	1.10 ± 0.49	0.13 to 2.07	2.250	0.052	1.40 ± 0.55	0.31 to 2.49	2.540	0.024
Release mechanics	0.007 ± 0.004	−0.001 to 0.015	1.720	0.086	0.80 ± 0.46	−0.11 to 1.71	1.740	0.084	0.90 ± 0.53	−0.15 to 1.95	1.700	0.091
Stability	0.013 ± 0.004	0.005 to 0.021	3.250	0.004	1.60 ± 0.50	0.61 to 2.59	3.200	0.008	1.70 ± 0.56	0.59 to 2.81	3.040	0.009

Note. B represents the adjusted regression coefficient, SE the standard error, and CI the confidence interval. Holm adjustment was applied separately across the four component-level comparisons for the MSEEM-feedback × Time effects, post-test contrasts, and retention contrasts.
